# Ammonium Salt‐Catalyzed Ring‐Opening of Aryl‐Aziridines with β‐Keto Esters

**DOI:** 10.1002/ejoc.202000916

**Published:** 2020-08-10

**Authors:** Victoria Haider, Viktoria Kreuzer, Maximilian Tiffner, Bernhard Spingler, Mario Waser

**Affiliations:** ^1^ Institute of Organic Chemistry Johannes Kepler University Linz Altenbergerstr. 69 4040 Linz Austria; ^2^ Department of Chemistry University of Zurich Winterthurerstrasse 190 8057 Zurich Switzerland

**Keywords:** Organocatalysis, Bifunctional ammonium salt, Aziridine opening, Alkylation, Regioselectivity

## Abstract

We herein report an ammonium salt‐catalyzed protocol for the regioselective ring opening of aryl‐aziridines with β‐keto esters. The reaction gives access to a variety of highly functionalized target molecules with two consecutive stereogenic centers and can be rendered enantioselective (up to *e.r*. = 91:9) by using bifunctional chiral ammonium salt catalysts.

## Graphical abstract

The quaternary ammonium salt‐catalyzed addition of β‐keto esters to aryl‐aziridines gives access to structurally diverse target molecules with two consecutive stereocenters.

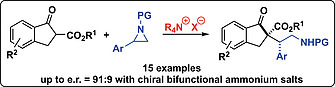

## Introduction

**Scheme 1:**
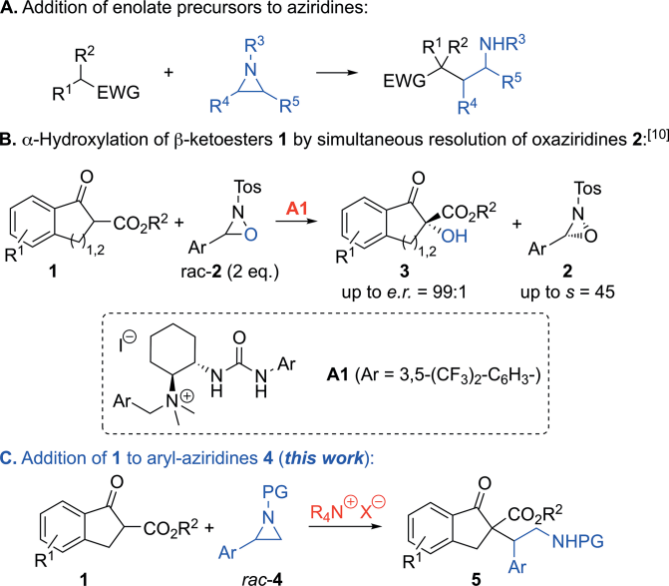
General reactivity of aziridines towards nucleophilic attack (A), our recently developed asymmetric α‐hydroxylation of β‐keto esters **1** with oxaziridines **2** (B), and the herein investigated addition of compounds **1** to aryl‐aziridines **4** (C).

Aziridines have been established as interesting building blocks for a variety of (asymmetric) transformations and their synthetic versatility can be attributed to the fact that they easily undergo ring opening reactions with a variety of different nucleophiles^1^ In addition, a broad variety of (chiral) aziridines can be accessed straightforwardly (racemic or enantiopure) by established synthesis strategies starting from simple precursors,^1^, ^2^ making approaches relying on aziridine opening reactions very appealing. The reactions of aziridines with C‐nucleophiles, i.e. enolate species, can lead to interesting γ‐amino‐carbonyl targets which are not easily accessible by other strategies. In addition, depending on the aziridine substitution pattern, rather complex structural motives containing two or even three consecutive stereogenic centers can be accessed (Scheme 1A) and the use of a chiral catalyst may allow to control such reactions in a stereoselective manner.^1^, ^3^, ^4^, ^5^, ^6^

Our group has a strong interest in asymmetric ion pairing catalysis^7^, ^8^, ^9^, ^10^ and we recently reported that bifunctional ammonium salt catalysts^8^ containing a (thio)‐urea H‐bonding motive (i.e. catalysts **A**) can be used for the asymmetric α‐hydroxylation of β‐keto esters **1** by using racemic oxaziridines **2** as the O‐transfer reagents (accompanied by a simultaneous resolution of the oxaziridines **2** as outlined in Scheme 1B).^10^ Based on the general interest in aziridine‐opening reactions and given our focus on asymmetric ammonium salt catalysis we now wanted to explore if ammonium salt catalysts (like compounds **A**) may also facilitate the addition of pronucleophiles **1** to aryl‐aziridines **4**. In general, the asymmetric ring‐opening of aziridines with β‐keto esters **1** using chiral ammonium salt catalysts has been well‐investigated, i.e. by the groups of Dixon and Jørgenson.^4^ However, in those cases no aryl‐aziridines **4** were used and to the best of our knowledge there are only very few reports in general, where enolate precursors were added to such aziridines in an asymmetric manner.^5^ Interestingly, some previous reports describing the addition of malonates to such aziridines in the absence of a chiral catalyst showed that these reagents predominately undergo nucleophile addition to the benzylic position of the aziridine, and that these reactions proceed via a stereospecific ring‐opening pathway with inversion of configuration of the benzylic stereogenic center.^6^ We thus wondered if we could introduce a protocol for the regio‐ and stereoselective addition of pronucleophiles **1** to (racemic) aziridines **4**, which would result in the formation of the highly functionalized target molecules **5** (Scheme 1C).

## Results and Discussion

We started our investigations by carrying out the reaction between the *tert*‐butyl ester **1a** and the phenyl aziridine **4a**. The reason why we opted for the *N*‐tosyl protected **4a** was because we previously (during the hydroxylation of **1** with oxaziridines **2**) realized that sulfonamide groups provide a very good point of coordination for our bifunctional ammonium salts **A**.^10^ Besides our own catalysts **A** 10, 11 (on which we put our main focus), we also compared the other classical and well‐established ammonium salt catalysts **B** 7, 12 and **C** 7, 13 and the recently reported bifunctional Cinchona alkaloid derivative **D**.^14^

First experiments without any catalyst (entry 1) and in the presence of a simple achiral ammonium salt (entry 2) clearly proved the beneficial effect of the phase‐transfer catalyst and showed that the reaction proceeds via addition to the benzylic carbon of the aziridine (giving **5a**). The relative configuration of the major diastereomer of product **5a** was assigned by single‐crystal X‐ray analysis^15^ and in all further experiments the *unlike* diastereomer was found to be the main product. We then carried out a first base screening with our urea‐containing catalyst **A1** (entries 3–6) and found that the targeted product **5a** could be obtained in a reasonable yield of 79 % and with promising enantio‐ and diastereoselectivities (*d.r*. = 6:1; *e.r*. = 89:11) after 24 h when using two equivalents of solid Cs_2_CO_3_ (entry 6). The use of aqueous bases on the other hand significantly slowed down the conversion (entry 4) and stronger bases (e.g. NaOH) were found to be not suited at all (results not given in the table ), i.e. as the aziridine decomposed significantly under these conditions. Interestingly, while the major diastereomer could be obtained with a good enantiomeric ratio of 89:11, the minor diastereomer was formed in an almost racemic manner (entry 6).

We then tested other derivatives of the bifunctional ammonium salts **A** (entries 7–9 give three representative examples), but neither the use of a thiourea (entry 7), nor using sterically more bulky systems (entry 9) resulted in any improvement. When using the well‐established Maruoka catalyst **B** next (entry 10),^7^, ^12^ the reaction stalled after around 50 % conversion and the observed diastereoselectivity was rather low (*d.r*. = 1.5:1). In sharp contrast to ammonium salt **A1** however, catalyst **B** allowed for higher enantioselectivities of the minor diastereomer (favoring the same major enantiomers of both diastereomers as **A1**). The classical Cinchona alkaloid ammonium salts **C** were found to be less suited for this reaction (entries 11 and 12), while the bifunctional catalyst **D** 14 gave at least some levels of selectivity (entry 13), although clearly not as promising as compounds **A**.

Based on these results, which support a beneficial effect of the bifunctional nature of the catalyst, we then tried to further optimize the reaction conditions using **A1** (entries 14–18). While non‐aromatic solvents were found to be less suited (entries 14, 15), the use of a lower amount of base resulted in a slightly increased enantioselectivity of 91:9 (compare entries 16 and 6). However, the yield was noticeable lower, and we found that actually this reaction stalled after around 60 % conversion, requiring addition of more base to proceed to completion. When carrying out the reaction under more concentrated conditions, the yield improved slightly, while the enantioselectivity decreased (entry 17). This result is in line with our recent observations for the α‐hydroxylation of compounds **1**, where higher dilution was beneficial for high enantioselectivities as well.^10^ Finally, lowering the catalyst loading to 5 mol‐% resulted in a slightly lower selectivity but in a noteworthy reduced yield. Again, the reaction stalled after a while and addition of more catalyst was necessary in those cases, while longer reaction times were not beneficial. This phenomenon was generally observed in all the other reactions that did not show a satisfying conversion after 24 h (compare with entries 4, 10, and 16). Lower temperatures were tried as well but the reactions became very slow with unreliable conversions and no benefit in enantioselectivity.

Unfortunately, during these optimization attempts we observed a very strong influence of the starting material quality on the stereoselectivity of the reaction. First, the aziridine **4** has to be rather clean and especially even very minor quantities of iodine residues originating from the synthesis route^16^ lead to a significant decrease of the *e.r*. In addition, the used β‐keto ester **1** has to be “perfectly clean” as well, as we observed that batches of **1a** that contained very small quantities of unidentified impurities (less than 1 % by ^1^H NMR) resulted in reduced enantioselectivities down to *e.r*. = 75:25 under otherwise identical conditions (in addition the yields were a bit higher here, demonstrating the notable influence of these unknown impurities on the reaction performance). Thus, use tests of each batch of the starting materials had to be made during all these optimizations. In sharp contrast to this pronounced sensitivity on the starting material quality observed for the synthesis of **5**, use tests of different keto ester **1a** batches for other reactions catalyzed by catalysts **A** (like the above mentioned α‐hydroxylation^10^ or analogous α‐halogenations^17^) revealed no measurable dependency of selectivity and/or yield therein. Accordingly, this rather unpractical sensitivity, where seemingly small impurities or changes in the reagent's quality affected the outcome significantly, required a tedious purification of the starting materials for all asymmetric experiments. As a consequence, the results given in entries 6 and 16 in Table 1 are the best we could obtain with carefully purified reagents only. On the other hand, it should be noted that with given qualities of a single batch of **1a** and/or **4a** reproducible results were obtained when repeating the experiments several times.

**Table 1:** Catalyst screening and optimization of the asymmetric reaction conditions^a^

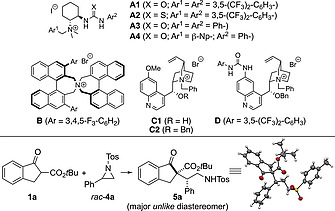						
Entry	Catalyst [mol‐%]	Solvent	Base	Yield [%]^b^	*d.r*.^c^	*e.r*._major_ d
1	–	toluene	K_2_CO_3_ (2 ×)	< 5	–	–
2	**TBAB** e (10 %)	toluene	K_2_CO_3_ (2 ×)	49	3:1	–
3	**A1** (10 %)	toluene	K_2_CO_3_ (2 ×)	66	3.5:1	85:15
4	**A1** (10 %)	toluene	K_2_CO_3_ (50 % aq.; 2 ×)	21^f^	4:1	86:14
5	**A1** (10 %)	toluene	K_2_HPO_4_ (2 ×)	71	2:1	76:24
6	**A1** (10 %)	toluene	Cs_2_CO_3_ (2 ×)	79	6:1	89:11 (56:44)^g^
7	**A2** (10 %)	toluene	Cs_2_CO_3_ (2 ×)	60	2.5:1	63:37
8	**A3** (10 %)	toluene	Cs_2_CO_3_ (2 ×)	72	1.5:1	81:19
9	**A4** (10 %)	toluene	Cs_2_CO_3_ (2 ×)	54	2:1	82:18
10	**B** (10 %)	toluene	Cs_2_CO_3_ (2 ×)	39^f^	1.5:1	70:30 (86:14)^g^
11	**C1** (10 %)	toluene	Cs_2_CO_3_ (2 ×)	89	2:1	52:48
12	**C2** (10 %)	toluene	Cs_2_CO_3_ (2 ×)	90	3:1	50:50
13	**D** (10 %)	toluene	Cs_2_CO_3_ (2 ×)	82	3:1	64:36
14	**A1** (10 %)	MTBE	Cs_2_CO_3_ (2 ×)	75	4:1	87:13
15	**A1** (10 %)	CH_2_Cl_2_	Cs_2_CO_3_ (2 ×)	84	3:1	66:34
16	**A1** (10 %)	toluene	Cs_2_CO_3_ (1 ×)	56	8:1	91:9
17	**A1** (10 %)	toluene^h^	Cs_2_CO_3_ (2 ×)	83	5:1	81:19
18	**A1** (5 %)	toluene	Cs_2_CO_3_ (2 ×)	36^f^	7:1	87:13

a All reactions were run at room temperature using 0.1 mmol **1a** and 0.2 mmol *rac*‐**4a** for 24 h in the indicated solvent (0.02 m with respect to **1a**) unless otherwise stated.

b Isolated yields of both diastereomers.

c Determined by ^1^H NMR and/or HPLC analysis of the crude product.

d Determined by HPLC using a chiral stationary phase.

e Tetrabutylammonium bromide.

f Less than 50–60 % conversion.

g *e.r*. of the minor diastereomer.

h 0.1 m with respect to **1a**.

During our **A1**‐catalyzed α‐hydroxylation of **1** with oxaziridines **2** we also observed a practical simultaneous kinetic resolution of compounds **2** (up to *s* = 45)^10^ and we therefore speculated that a resolution may be possible for our herein used aziridine **4a** too. We thus carried out the addition of **1a** to *rac*‐**4a** (2 equiv.) under the optimized conditions (entry 16, Table 1) and observed some moderate enantioenrichment of recovered **4a** as well [*e.r*. = 60:40 for (*S*)‐**4a** after 25 % conversion; *s* = 4.8]. Giving the significantly lower stereoselectivity in the synthesis of **5a** compared to our previous synthesis of **3**, this lower efficiency for the simultaneous resolution of **4a** comes as no surprise, but nevertheless this result supports our initial hypothesis.

**Scheme 2:**
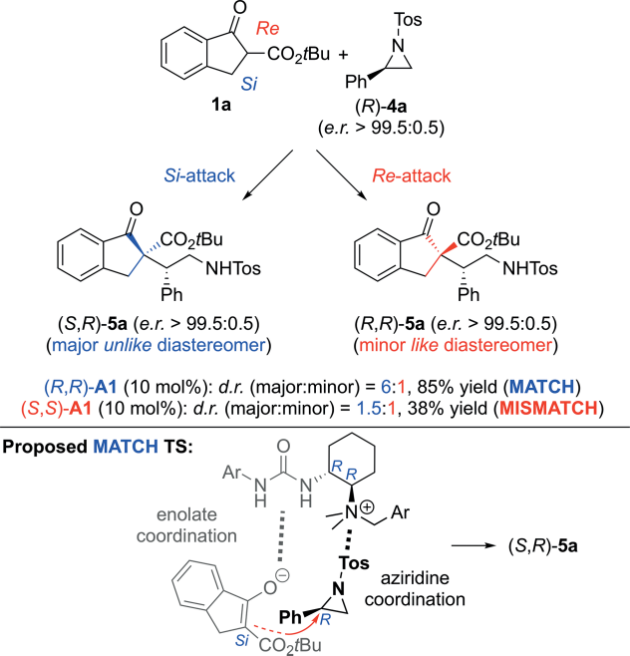
Match/mismatch scenario in the addition of **1a** to (*R*)‐**4a** in the presence of both enantiomers of the catalyst **A1** and the proposed match‐transition state.

To gather further information about this aziridine‐opening reaction, we next carried out the addition of **1a** to enantiopure (*R*)‐**4a** 18 in the presence of either (*R,R*)‐**A1** or (*S,S*)‐**A1** as a catalyst (Scheme 2).

In both cases, the two diastereomers of **5a** were formed with complete enantiospecifity, supporting an S_N_2‐mechansim for the ring opening, as observed by others as well.^6^ The use of the (*R,R*)‐catalyst enantiomer (which was also used during the screening summarized in Table 1) lead to a reasonable diastereoselectivity of 6:1 in favor of the major *unlike* diastereomer with high yield (match case). On the other hand, the reaction with the (*S,S*)‐catalyst proceeded significantly slower and with much lower diastereoselectivity (mismatch case). In our previous investigations on the use of catalysts **A** for reactions of β‐keto esters **1** we observed that the (*S,S*)‐catalyst enantiomers always favor *Re*‐face addition of the nucleophile.^10^, ^17^ In addition, previous DFT calculations of the **A1**‐catalyzed α‐hydroxylation of **1a** with **2** support a transition state where the enolate is H‐bonded to the urea moiety, while the electrophile is coordinated to the ammonium group.^10^, ^19^ Based on these previous observations, the herein observed pronounced match/mismatch behavior in the stereospecific addition to enantiopure aziridine **4a**, and the unambiguously determined relative configuration of **4a**,^15^ the (*R,R*)‐catalyst is supposed to interact with both starting materials **1a** and **4a** in an organized bifunctional manner as illustrated in Scheme 2, rationalizing formation of the observed favored major stereoisomer (*S,R*)‐**5a**. Based on this model it can also be proposed that the slightly preferred enantiomer of the minor *like* diastereomer is (*S,S*)‐**5a**.

Summing these investigations up, it was demonstrated that the stereoselective addition of pronucleophile **1a** to aziridine **4a** can be controlled by using the bifunctional ammonium salts **A**. Unfortunately however, because of the unexpected pronounced sensitivity that we observed during all these test reactions, and despite the promising selectivities up to *e.r*. = 91:9, this reaction as such is far from being as robust and practical as other chiral ammonium salt catalyzed reactions that we investigated recently.^9^, ^10^, ^17^

Considering the novelty of the products **5** that are accessible by this strategy in general, but keeping in mind the practical limitations of the asymmetric protocol, i.e. the sensitivity to the starting material qualities, we thus investigated the application scope for the racemic phase transfer‐catalyzed addition of various β‐keto esters **1** to aryl‐aziridines **4** only.

**Scheme 3:**
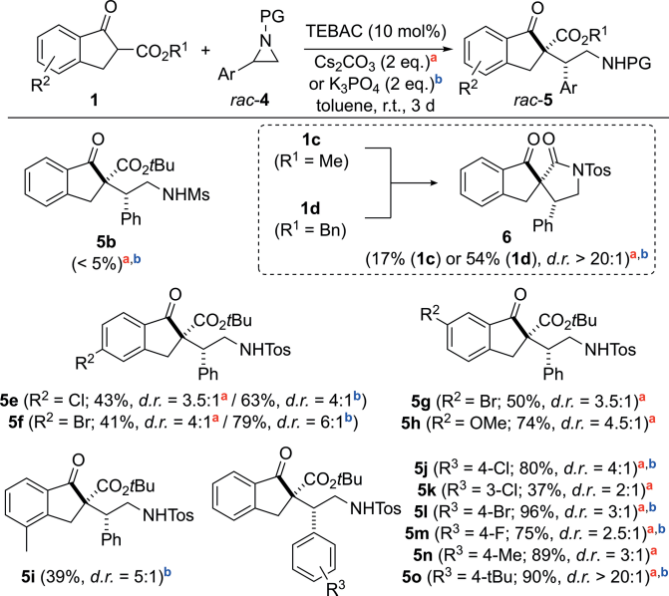
Application scope of the racemic ammonium salt‐catalyzed ring‐opening of aryl‐aziridines **4** with β‐keto esters **1**.

As shown in Scheme 3, a variety of differently functionalized pronucleophiles **1** and acceptors **4** were reacted for three days in the presence of benzyltriethylammonium chloride (TEBAC) using Cs_2_CO_3_ (a) or K_3_PO_4_ (b) as the base. Interestingly, mesylated aziridines did not react well (see the result given for product **5b**) and when using *N*‐Boc aziridines no reaction was observed at all. On the other hand, the use of methyl or benzyl esters revealed a strong influence of this group on the overall outcome, as both of them resulted in the direct formation of the spirocyclic γ‐lactam **6** upon addition to aziridine **4a**. Different substituents on the donor side were tolerated (see products **5e**–**i**), although in some of these cases the use of K_3_PO_4_ was found to be beneficial over Cs_2_CO_3_ (in the latter case significant amounts of decomposition products were observed). On the other hand, variations of the acceptor aryl group were generally well accepted in the 4‐position, independent of the nature of the base, while the 3‐position was lower yielding (see product **55**) and in this case again significant amounts of unidentified decomposition products were formed. Nevertheless, the racemic protocol generally gives access to a variety of differently functionalized products **5** with relatively good yields and reasonable diastereoselectivities under these operationally simple ammonium salt‐catalyzed conditions.

## Conclusions

Summing these investigations up, we have shown that β‐keto esters **1** react with aryl‐aziridines **4** under (chiral) ammonium salt catalysis giving access to highly functionalized products **5**. In principle a catalytic and reasonably enantioselective protocol could be developed by using the bifunctional ammonium salt catalysts **A**. Unfortunately, however, this reaction was found to be very sensitive towards the quality of the starting materials. Nevertheless, control experiments showed a pronounced match/mismatch behavior of the bifunctional catalyst and the used aziridine, thus pointing towards a mechanism where the catalyst simultaneously activates both of the reaction partners.

## Experimental Section

General experimental and analytical details as well as the characterization data of all the novel compounds can be found in the online supporting information.

**Racemic Protocol:** A mixture of 0.1 mmol β‐keto ester **1** (1 equiv.), 2.2 mg of TEBAC (0.01 mmol, 10 mol‐%) and 0.2 mmol base (2 equiv.) was dissolved in 5 mL of toluene (Ar‐atmosphere). Then 0.2 mmol of aziridine **4** (2 equiv.) were added in one portion. After a reaction time of three days the mixture was filtered through a plug of Na_2_SO_4_ and washed with DCM. The crude product, obtained after evaporation of the solvent, was subjected to column chromatography (silica gel, heptanes:EtOAc = 5:1) to isolate products **5** and **6** as mixtures of diastereomers. The diastereomers were in some cases separated using preparative HPLC (Grace Alltima Silica 10 µm 250 × 10 mm, *n*‐hexane/EtOAc, 5 mL/min).

**Enantioselective Screening Protocol:** A mixture of the β‐keto ester **1a** (0.1 mmol, 1 equiv.), catalyst **A1** (7.6 mg, 0.01 mmol, 10 mol‐%) and 0.2 mmol of the tested base (2 equiv.) were dissolved in 5 mL of the given solvent (argon atmosphere, room temperature). Then 48 mg of 2‐phenyltosylaziridine **4a** (0.2 mmol, 2 equiv.) were added in one portion. After a reaction time of one day the mixture was filtered through a plug of Na_2_SO_4_ and washed with DCM. The crude product, obtained after evaporation of the solvent under reduced pressure, was then subjected to column chromatography purification (silica gel, heptanes:EtOAc = 2:1) to isolate product **5a** as a mixture of two diastereomers (*d.r*. up to 8:1) in yields up to 84 %. The diastereomers were separated using preparative HPLC (Grace Alltima Silica 10 µm 250 × 10 mm, *n*‐hexane/EtOAc = 9:1, 5 mL/min, retention times: 39.1 min major, 46.8 min minor). The enantiomeric excess of the minor diastereomer was determined by HPLC using a YMC Amylose SA column (*n*‐hexane/*i*PrOH = 3:1, 1 mL/min, 10 °C, retention times: 21.0 and 35.5 min). The enantiomeric excess of the major diastereomer was determined by HPLC using a YMC Cellulose SB column (*n*‐hexane/*i*PrOH = 10:1, 1 mL/min, 10 °C, retention times: 22.2 min major, 20.2 min minor).

**Analytical Details for Compound 5a:** HRMS (ESI): *m/z* calculated for C_29_H_32_NO_5_S^+^: 506.2001 [M + H]^+^, found 506.1992. *Major Diastereomer*: ^1^H‐NMR (700 MHz, CDCl_3_, 298.0 K): *δ* /ppm = 7.67 (d, *J* = 8.2 Hz, 2H), 7.49 (d, *J* = 7.7 Hz, 1H), 7.46 (t, *J* = 7.2 Hz, 1H), 7.32 (d, *J* = 7.7 Hz, 1H), 7.28 (d, *J* = 8.2 Hz, 2H), 7.21 (t, *J* = 7.2 Hz, 1H), 7.11–7.05 (m, 5H), 4.42–4.40 (m, 1H), 3.87 (dd, *J*_1_ = 9.7 Hz, *J*_2_ = 5.7 Hz, 1H), 3.69 (d, *J* = 17.2 Hz, 1H), 3.65–3.61 (m, 1H), 3.38–3.34 (m, 1H), 3.22 (d, *J* = 17.2 Hz, 1H), 2.43 (s, 3H), 1.37 (s, 9H); ^13^C‐NMR (176 MHz, CDCl_3_, 298.0 K): *δ* /ppm = 200.7, 169.0, 153.2, 143.6, 137.2, 136.2, 135.2, 135.0, 129.9, 129.6, 128.7, 127.8, 127.6, 127.3, 126.0, 124.7, 83.1, 65.4, 48.0, 44.4, 33.3, 27.8, 21.6. *Minor Diastereomer*: ^1^H‐NMR (700 MHz, CDCl_3_, 298.0 K): *δ* /ppm = 7.73 (d, *J* = 7.5 Hz, 2H), 7.56 (d, *J* = 8.1 Hz, 2H), 7.56–7.54 (m, 1H), 7.37–7.34 (m, 2H), 7.22–7.18 (m, 5H), 7.11–7.08 (m, 2H), 4.20–4.18 (m, 1H), 3.90–3.88 (m, 1H), 3.64 (d, *J* = 16.8 Hz, 1H), 3.40–3.36 (m, 1H), 3.33–3.29 (m, 1H), 3.27 (d, *J* = 16.8 Hz, 1H), 2.40 (s, 3H), 1.20 (s, 9H); ^13^C‐NMR (176 MHz, CDCl_3_, 298.0 K): *δ* /ppm = 202.1, 168.5, 153.2, 143.4, 137.9, 137.0, 135.7, 135.4, 129.8, 129.2, 128.8, 127.9, 127.8, 127.2, 126.3, 124.7, 82.8, 65.2, 49.0, 45.5, 34.7, 27.6, 21.6.

## Supporting information

Supporting Information
