# Supplementary material for: Ammonium Salt‐Catalyzed Ring‐Opening of Aryl‐Aziridines with β‐Keto Esters
Source: European J Org Chem. 2020 Aug 10;2020(32):5173–7. doi: 10.1002/ejoc.202000916 (PMC7508174; doi:10.1002/ejoc.202000916)
Supplement: Supplementary file 1 — Supporting Information [file EJOC-2020-5173-s001.pdf]

European Journal of Organic Chemistry

Supporting Information

Ammonium Salt-Catalyzed Ring-Opening of Aryl-Aziridines with β-Keto Esters

Victoria Haider, Viktoria Kreuzer, Maximilian Tiffner, Bernhard Spingler, Mario Waser*

1. GENERAL INFORMATION......2

1.1. GENERAL METHODS......2

1.2. SINGLE-CRYSTAL ANALYSIS......3

2. EXPERIMENTAL PROCEDURES......16

2.1. GENERAL PROCEDURES FOR THE RING-OPENING REACTIONS OF ARYL-AZIRIDINES 4 WITH Β-KETOESTERS 1......16

2.1.1. GENERAL PROCEDURE FOR THE ENANTIOSELECTIVE SCREENING AND OPTIMIZATION REACTIONS......16

2.1.2. GENERAL PROCEDURE FOR THE RACEMIC APPLICATION SCOPE......17

3. ILLUSTRATIVE NMR SPECTRA OF NEW COMPOUNDS......26

4. HPLC TRACES......54

1. General Information

1.1. General Methods

Solvents and reagents were purchased from commercial suppliers and were used without further purification unless otherwise mentioned. Anhydrous solvents were dried using a molecular sieve drying plant. All reactions were performed under an argon atmosphere. TLC analysis were carried out using Macherey-Nagel pre-coated TLC sheets Alugram ® Xtra SIL G/UV with detection at 254 nm. For column chromatography Davisil LC 60A 70-200 MICRON silica gel was used. HPLC was performed using a Dionex Summit HPLC system with a YMC Chiral Art Cellulose - SB (250 x 4.6 mm, 5 µm) and a YMC Chiral Art Amylose SA (250 x 4.6 mm, 5 µm) chiral stationary phase. Preparative HPLC was carried out using a Thermo Scientific Dionex Ultimate 3000 system with variable wavelength detection and a Grace Alltima Silica 10µm 250x10 mm column. ^{1}H- and ^{13}C-NMR spectra were recorded using a Bruker Avance III 300 MHz spectrometer with a broad band observe probe and a sample changer for 16 samples, a Bruker Avance DRX 500 MHz spectrometer, and a Bruker Avance III 700 MHz spectrometer with an Ascend magnet and TCI cryoprobe of the Austro-Czech NMR-Research Center. High resolution mass spectra were obtained using a Thermo Fisher Scientific LTQ Orbitrap XL with an Ion Max API Source.

Chiral catalysts **A**[1], starting β-ketoesters **1**[2], and racemic[3] and enantiopure[4] aziridines **4** were synthesized as described previously.

---

1 M. Tiffner, J. Novacek, A. Busillo, K. Gratzer, A. Massa, M. Waser, *RSC Adv*. **2015**, *5*, 78941-78949.

2 a) Y.-N. Duan, L.-Q. Cui, L.-H. Zuo, C. Zhang, *Chem. Eur. J.* **2015**, *21*, 13052-13057; b) X. Gu, Y. Zhang, Z.-J. Xu, C.-M. Che, *Chem. Commun*. **2014**, *50*, 7870-7873; c) I. Geibel, J. Christoffers, *Eur. J. Org. Chem*. **2016**, 918-920; d) A. M. R. Smith, H. S. Rzepa, A. J. P. White, D. Billen, K. K. M. Hii, *J. Org. Chem.* **2010**, *75*, 3085-3096.

3 H. Rubin, J. Cockrell, J. B. Morgan, *J. Org. Chem*. **2013**, *78*, 8865-8871.

4 R. A. Craig, N. R. O'Connor, A. F. G. Goldberg, B. M. Stoltz, *Chem. Eur. J*. **2014**, *20*, 4806-4813.

1.2. Single-Crystal Analysis

Crystallographic data were collected at 160.0(1) K on a Rigaku-Oxford Diffraction XtaLAB Synergy-S dual source diffractometer. This is a kappa-axis four-circle goniometer with a Dectris Pilatus3 R 200K HPC (Hybrid Photon Counting) detector and Cu and Mo PhotonJet microfocus X-ray sources. A suitable crystal was covered with oil (Infineum V8512, formerly known as Paratone N), placed on a nylon loop that is mounted on a CrystalCap Magnetic™ pin (Hampton Research) and immediately transferred to the diffractometer. The program suite *CrysAlis^{Pro}* was used for data collection, numerical and multi-scan absorption correction as well as data reduction.[5] The structure was solved with the dual-space algorithm using *SHELXT*[6] and was refined by full-matrix least-squares methods on F^2 with *SHELXL-2018*[7] using the *Olex2* GUI.[8] Compound **5a** crystallized in the chiral space group *C*2 with a Flack parameter of 0.011(6) (*S* configuration at C1 and *R* configuration at C15). The sulfonylamide methylene unit was disordered in a ratio 78:22. The graphical output was produced with the help of the program *Mercury*.[9[5]] CCDC 2012449 contains the supplementary crystallographic data for this paper. These data are provided free of charge by The Cambridge Crystallographic Data Centre *via* www.ccdc.cam.ac.uk/structures.

---

[5] *CrysAlis^{Pro} Software system*; Rigaku Oxford Diffraction, vers. 1.171.40; Rigaku Corporation, 2019.
[6] G. M. Sheldrick, *Acta Cryst.* **2015**, *A71*, 3.
[7] G. M. Sheldrick, *Acta Cryst.* **2015**, *C71*, 3.
[8] O. V. Dolomanov, L. J. Bourhis, R. J. Gildea, J. A. K. Howard, H. Puschmann, *J. Appl. Cryst.* **2009**, *42*, 339.
[9] C. F. Macrae, L. Sovago, S. J. Cottrell, P. T. A. Galek, P. McCabe, E. Pidcock, M. Platings, G. P. Shields, J. S. Stevens, M. Towler, P. A. Wood, *J. Appl. Cryst.* **2020**, *53*, 226.

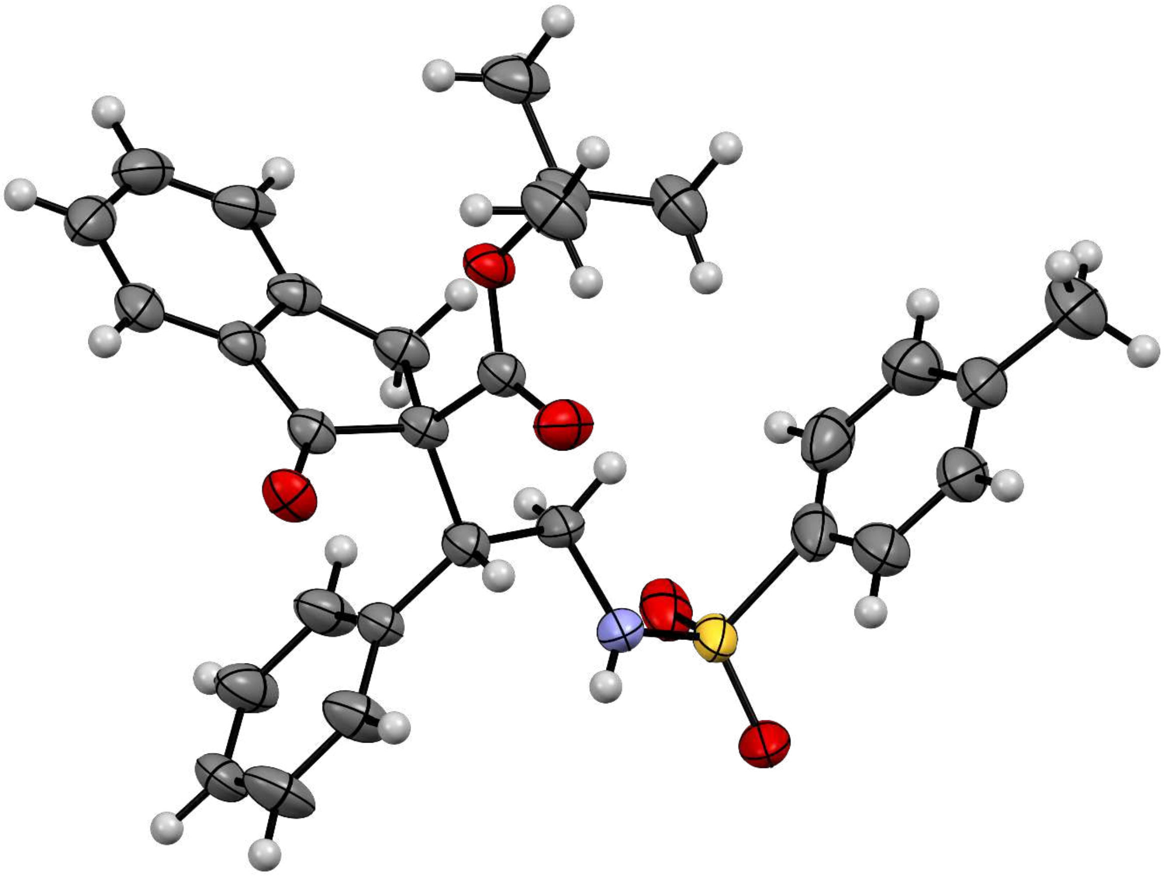

Fig. S1: Displacement ellipsoid representation of compound **5a** at 50% probability. Disordered parts are omitted for clarity.

Table S1. Crystal data and structure refinement for **5a**.

Empirical formula	$C_{29}H_{31}NO_5S$
Formula weight	505.61
Crystal system	Monoclinic
Space group	C2
a [Å]	20.65539(16)
b [Å]	5.88663(4)
c [Å]	22.57135(19)
α [°]	90
β [°]	103.4741(9)
γ [°]	90
Volume [$Å^3$]	2668.92(4)
Z	4
Density (calculated) [Mg/m^3]	1.258
Temperature [K]	160.01(10)
Wavelength [Å]	1.54184
Absorption coefficient [mm^{-1}]	1.392
F(000)	1072
Crystal size [mm^3]	0.594 x 0.122 x 0.101
Crystal description	colourless needle
Theta range for data collection [°]	4.028 to 79.558
Index ranges	-21<=h<=26, -7<=k<=7, -27<=l<=28
Reflections collected	31300
Independent reflections	5436 [R(int) = 0.0260]
Reflections observed	5380
Criterion for observation	$I > 2 \sigma (I)$
Completeness to theta	94.4 % to 67.684°
Absorption correction	Gaussian
Max. and min. transmission	1.000 and 0.344
Data / restraints / parameters	5436 / 98 / 375
Goodness-of-fit on F^2	1.057
Final R indices [$I > 2 \sigma (I)$]	R1 = 0.0359, wR2 = 0.0973
R indices (all data)	R1 = 0.0365, wR2 = 0.0997
Absolute structure parameter	0.011(6)
Largest diff. peak and hole [$e.Å^{-3}$]	0.249 and -0.200

Table S2. Atomic coordinates (x 10^4) and equivalent isotropic displacement parameters ($Å^2$x 10^3) for **5a**. U(eq) is defined as one third of the trace of the orthogonalized U^{ij} tensor.

	x	y	z	U(eq)
S(1)	8205(1)	2033(3)	6408(1)	47(1)
S(1B)	8032(2)	1079(8)	6507(2)	35(1)
O(1)	7625(1)	8928(3)	8714(1)	41(1)
O(2)	6758(1)	8461(3)	7220(1)	50(1)
O(3)	6062(1)	6663(3)	7694(1)	42(1)
O(4)	8702(1)	2763(6)	6099(1)	59(1)
O(4B)	8568(6)	2419(15)	6344(5)	40(2)
O(5)	8269(2)	-115(7)	6715(1)	56(1)
O(5B)	8137(6)	-1327(19)	6624(4)	44(2)
N(1)	8190(1)	4008(6)	6910(1)	46(1)
N(1B)	7887(4)	2274(17)	7123(4)	39(2)
C(1)	7199(1)	5772(4)	8029(1)	33(1)
C(2)	7325(1)	7146(4)	8630(1)	32(1)
C(3)	7005(1)	5926(4)	9056(1)	34(1)
C(4)	6922(1)	6627(5)	9621(1)	44(1)
C(5)	6588(1)	5176(6)	9934(1)	55(1)
C(6)	6346(1)	3104(6)	9681(1)	57(1)
C(7)	6430(1)	2420(5)	9123(1)	48(1)
C(8)	6764(1)	3858(4)	8803(1)	37(1)
C(9)	6933(1)	3458(4)	8199(1)	37(1)
C(10)	6655(1)	7126(4)	7592(1)	35(1)
C(11)	5430(1)	7480(4)	7291(1)	41(1)
C(12)	4920(1)	6420(6)	7596(2)	58(1)
C(13)	5377(2)	6555(5)	6660(1)	55(1)
C(14)	5390(2)	10036(5)	7306(2)	56(1)
C(15)	7830(1)	5691(4)	7766(1)	38(1)
C(16)	8482(1)	5315(4)	8225(1)	36(1)
C(17)	8643(1)	3348(5)	8560(1)	51(1)
C(18)	9247(1)	3114(6)	8979(2)	59(1)
C(19)	9705(1)	4849(5)	9072(1)	54(1)
C(20)	9560(1)	6788(5)	8729(2)	61(1)
C(21)	8959(1)	7018(5)	8315(1)	53(1)
C(22)	7686(2)	3834(7)	7272(2)	42(1)
C(22B)	7805(6)	4770(20)	7083(6)	38(2)

C(23)	7408(1)	1936(6)	5885(1)	55(1)
C(24)	7269(1)	3756(6)	5487(1)	58(1)
C(25)	6678(1)	3796(6)	5053(1)	56(1)
C(26)	6219(1)	2023(6)	4991(1)	55(1)
C(27)	6366(2)	242(7)	5402(2)	63(1)
C(28)	6954(2)	198(7)	5853(1)	64(1)
C(29)	5604(2)	2049(9)	4486(2)	82(1)

Table S3. Bond lengths [Å] and angles [°] for **5a**.

S(1)-O(4)	1.435(3)
S(1)-O(5)	1.433(4)
S(1)-N(1)	1.629(3)
S(1)-C(23)	1.789(3)
S(1B)-O(4B)	1.475(10)
S(1B)-O(5B)	1.448(10)
S(1B)-N(1B)	1.647(10)
S(1B)-C(23)	1.744(4)
O(1)-C(2)	1.211(3)
O(2)-C(10)	1.206(3)
O(3)-C(10)	1.326(3)
O(3)-C(11)	1.486(3)
N(1)-C(22)	1.468(4)
N(1B)-C(22B)	1.480(17)
C(1)-C(2)	1.548(3)
C(1)-C(9)	1.549(3)
C(1)-C(10)	1.534(3)
C(1)-C(15)	1.554(3)
C(2)-C(3)	1.472(3)
C(3)-C(4)	1.390(3)
C(3)-C(8)	1.387(3)
C(4)-C(5)	1.390(4)
C(5)-C(6)	1.391(5)
C(6)-C(7)	1.371(4)
C(7)-C(8)	1.396(3)
C(8)-C(9)	1.502(3)
C(11)-C(12)	1.520(4)
C(11)-C(13)	1.506(4)
C(11)-C(14)	1.507(4)
C(15)-C(16)	1.513(3)
C(15)-C(22)	1.541(4)
C(15)-C(22B)	1.625(12)
C(16)-C(17)	1.380(3)
C(16)-C(21)	1.386(3)
C(17)-C(18)	1.386(4)
C(18)-C(19)	1.375(4)
C(19)-C(20)	1.372(4)

C(20)-C(21)	1.376(4)
C(23)-C(24)	1.384(5)
C(23)-C(28)	1.378(5)
C(24)-C(25)	1.377(4)
C(25)-C(26)	1.396(5)
C(26)-C(27)	1.386(5)
C(26)-C(29)	1.496(4)
C(27)-C(28)	1.391(5)
O(4)-S(1)-N(1)	104.9(2)
O(4)-S(1)-C(23)	110.01(16)
O(5)-S(1)-O(4)	120.24(19)
O(5)-S(1)-N(1)	107.9(2)
O(5)-S(1)-C(23)	104.9(2)
N(1)-S(1)-C(23)	108.46(14)
O(4B)-S(1B)-N(1B)	106.1(6)
O(4B)-S(1B)-C(23)	95.8(4)
O(5B)-S(1B)-O(4B)	118.6(6)
O(5B)-S(1B)-N(1B)	108.2(6)
O(5B)-S(1B)-C(23)	118.7(5)
N(1B)-S(1B)-C(23)	108.1(4)
C(10)-O(3)-C(11)	122.85(17)
C(22)-N(1)-S(1)	118.0(3)
C(22B)-N(1B)-S(1B)	114.3(8)
C(2)-C(1)-C(9)	103.85(17)
C(2)-C(1)-C(15)	111.51(17)
C(9)-C(1)-C(15)	116.38(17)
C(10)-C(1)-C(2)	103.90(16)
C(10)-C(1)-C(9)	111.54(18)
C(10)-C(1)-C(15)	108.89(18)
O(1)-C(2)-C(1)	124.37(19)
O(1)-C(2)-C(3)	127.7(2)
C(3)-C(2)-C(1)	107.89(18)
C(4)-C(3)-C(2)	128.6(2)
C(8)-C(3)-C(2)	109.42(19)
C(8)-C(3)-C(4)	122.0(2)
C(5)-C(4)-C(3)	117.8(3)
C(4)-C(5)-C(6)	120.3(3)
C(7)-C(6)-C(5)	121.7(2)

C(6)-C(7)-C(8)	118.8(3)
C(3)-C(8)-C(7)	119.5(2)
C(3)-C(8)-C(9)	111.93(18)
C(7)-C(8)-C(9)	128.5(2)
C(8)-C(9)-C(1)	104.91(18)
O(2)-C(10)-O(3)	125.1(2)
O(2)-C(10)-C(1)	124.4(2)
O(3)-C(10)-C(1)	110.44(17)
O(3)-C(11)-C(12)	101.17(19)
O(3)-C(11)-C(13)	108.80(19)
O(3)-C(11)-C(14)	110.8(2)
C(13)-C(11)-C(12)	112.0(2)
C(13)-C(11)-C(14)	112.9(2)
C(14)-C(11)-C(12)	110.6(2)
C(1)-C(15)-C(22B)	122.0(5)
C(16)-C(15)-C(1)	115.84(19)
C(16)-C(15)-C(22)	112.5(2)
C(16)-C(15)-C(22B)	115.3(4)
C(22)-C(15)-C(1)	105.2(2)
C(17)-C(16)-C(15)	124.0(2)
C(17)-C(16)-C(21)	117.0(2)
C(21)-C(16)-C(15)	118.9(2)
C(16)-C(17)-C(18)	121.3(2)
C(19)-C(18)-C(17)	120.6(3)
C(20)-C(19)-C(18)	118.7(3)
C(19)-C(20)-C(21)	120.5(3)
C(20)-C(21)-C(16)	121.8(3)
N(1)-C(22)-C(15)	108.2(3)
N(1B)-C(22B)-C(15)	107.1(9)
C(24)-C(23)-S(1)	115.0(3)
C(24)-C(23)-S(1B)	138.4(3)
C(28)-C(23)-S(1)	124.7(3)
C(28)-C(23)-S(1B)	101.2(3)
C(28)-C(23)-C(24)	120.3(3)
C(25)-C(24)-C(23)	119.4(3)
C(24)-C(25)-C(26)	121.8(3)
C(25)-C(26)-C(29)	120.3(3)
C(27)-C(26)-C(25)	117.5(3)
C(27)-C(26)-C(29)	122.2(3)

C(26)-C(27)-C(28)	121.4(3)
C(23)-C(28)-C(27)	119.5(3)

Symmetry transformations used to generate equivalent atoms:

Table S4. Anisotropic displacement parameters ($Å^2 x 10^3$) for **5a**. The anisotropic displacement factor exponent takes the form: $-2\pi^2[h^2 a^{*2}U^{11} + ... + 2 h k a^* b^* U^{12}]$

	U^{11}	U^{22}	U^{33}	U^{23}	U^{13}	U^{12}
S(1)	30(1)	80(1)	31(1)	-6(1)	5(1)	9(1)
S(1B)	31(2)	45(2)	31(2)	2(1)	12(1)	1(2)
O(1)	44(1)	30(1)	47(1)	0(1)	3(1)	-4(1)
O(2)	39(1)	58(1)	54(1)	22(1)	10(1)	4(1)
O(3)	26(1)	52(1)	43(1)	13(1)	1(1)	2(1)
O(4)	33(1)	106(2)	41(2)	-9(2)	13(1)	4(1)
O(4B)	28(5)	55(5)	41(6)	10(4)	17(4)	-10(4)
O(5)	43(2)	77(2)	44(2)	-2(2)	0(1)	27(2)
O(5B)	60(6)	40(5)	36(4)	0(4)	20(4)	10(4)
N(1)	31(1)	73(2)	34(1)	-7(1)	10(1)	-4(1)
N(1B)	35(4)	50(4)	32(3)	8(3)	10(3)	0(3)
C(1)	28(1)	31(1)	38(1)	1(1)	4(1)	0(1)
C(2)	28(1)	29(1)	37(1)	5(1)	1(1)	4(1)
C(3)	24(1)	39(1)	37(1)	8(1)	1(1)	5(1)
C(4)	32(1)	57(1)	39(1)	4(1)	1(1)	7(1)
C(5)	36(1)	88(2)	42(1)	15(1)	10(1)	10(1)
C(6)	34(1)	80(2)	57(2)	27(1)	10(1)	-3(1)
C(7)	29(1)	49(1)	64(2)	21(1)	4(1)	-3(1)
C(8)	24(1)	38(1)	47(1)	12(1)	1(1)	3(1)
C(9)	31(1)	29(1)	49(1)	1(1)	4(1)	-1(1)
C(10)	30(1)	36(1)	36(1)	2(1)	3(1)	-1(1)
C(11)	27(1)	43(1)	46(1)	3(1)	-5(1)	2(1)
C(12)	30(1)	65(2)	74(2)	14(1)	6(1)	2(1)
C(13)	46(1)	58(2)	54(2)	-8(1)	-5(1)	2(1)
C(14)	47(2)	42(1)	70(2)	-2(1)	-3(1)	4(1)
C(15)	30(1)	45(1)	37(1)	-2(1)	5(1)	1(1)
C(16)	28(1)	41(1)	36(1)	-3(1)	5(1)	-3(1)
C(17)	32(1)	54(1)	62(2)	14(1)	-1(1)	-10(1)
C(18)	36(1)	73(2)	63(2)	29(2)	-1(1)	-6(1)
C(19)	29(1)	77(2)	48(1)	4(1)	-5(1)	-8(1)
C(20)	34(1)	55(2)	85(2)	2(2)	-6(2)	-13(1)
C(21)	36(1)	43(1)	73(2)	8(1)	-1(1)	-6(1)
C(22)	29(1)	60(2)	38(2)	-9(2)	12(1)	-2(1)
C(22B)	29(4)	53(5)	34(4)	5(4)	9(4)	-2(4)

C(23)	43(1)	88(2)	32(1)	-7(1)	7(1)	18(1)
C(24)	39(1)	80(2)	52(2)	-3(1)	5(1)	3(1)
C(25)	42(1)	81(2)	42(1)	16(1)	4(1)	6(1)
C(26)	38(1)	86(2)	41(1)	7(1)	5(1)	5(1)
C(27)	48(2)	80(2)	59(2)	11(2)	10(2)	-1(2)
C(28)	59(2)	88(2)	44(2)	19(2)	13(2)	21(2)
C(29)	48(2)	125(3)	62(2)	11(2)	-5(2)	4(2)

Table S5. Hydrogen coordinates (x 10^4) and isotropic displacement parameters ($Å^2$x 10^3) for **5a**.

	x	y	z	U(eq)
H(1)	8474	5144	6963	55
H(1B)	7860	1493	7449	47
H(4)	7088	8052	9788	52
H(5)	6526	5602	10323	66
H(6)	6115	2138	9900	69
H(7)	6264	993	8958	58
H(9A)	6534	2990	7888	45
H(9B)	7278	2264	8234	45
H(12A)	4966	4763	7597	86
H(12B)	4471	6842	7370	86
H(12C)	4993	6972	8016	86
H(13A)	5714	7276	6480	83
H(13B)	4933	6879	6407	83
H(13C)	5450	4909	6681	83
H(14A)	5487	10556	7730	84
H(14B)	4942	10525	7096	84
H(14C)	5716	10687	7100	84
H(15)	7861	7175	7559	45
H(15A)	7875	7353	7693	45
H(17)	8333	2129	8502	62
H(18)	9345	1744	9204	71
H(19)	10114	4708	9367	64
H(20)	9878	7980	8777	74
H(21)	8868	8381	8086	63
H(22A)	7707	2314	7464	50
H(22B)	7236	4045	7007	50
H(22C)	7374	5169	6805	46
H(22D)	8167	5464	6924	46
H(24)	7579	4965	5514	69
H(25)	6580	5067	4787	67
H(27)	6058	-974	5376	75
H(28)	7042	-1021	6136	76
H(29A)	5302	3239	4563	122

H(29B)	5382	570	4464	122
H(29C)	5725	2354	4099	122

2. Experimental procedures

2.1. General procedures for the ring-opening reactions of aryl-aziridines 4 with β-ketoesters 1

2.1.1. General procedure for the enantioselective screening and optimization reactions

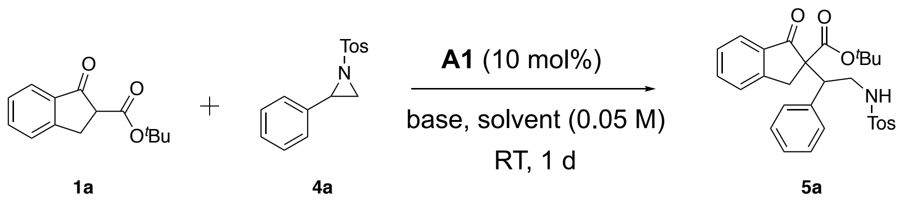

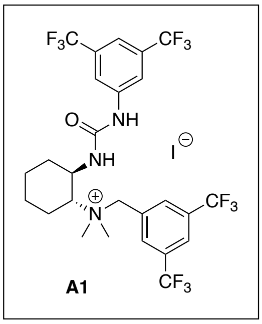

A mixture of the β-ketoester **1a** (0.1 mmol, 1 equiv.), catalyst **A1** (7.6 mg 0.01 mmol, 10 mol%) and 0.2 mmol of the tested base (2 equiv.) was dissolved in 5 mL of the given solvent under an argon atmosphere (room temperature). Then 48 mg of 2-phenyltosylaziridine **4a** (0.2 mmol, 2 equiv.) were added in one portion. After a reaction time of one day the mixture was filtrated through a plug of Na_2SO_4 and washed with DCM. The crude product, obtained after evaporation of the solvent under reduced pressure, was then subjected to column chromatography purification (silica gel, heptanes:EtOAc = 2:1) to isolate product **5a** as a mixture of two diastereomers (d.r. up to 8:1) in yields up to 84%. The diastereomers were separated using preparative HPLC (Grace Alltima Silica 10 µm 250x10 mm, n-hexane:EtOAc = 9:1, 5 mL/min, retention times: 39.1 min major, 46.8 min minor). The enantiomeric excess of the minor diastereomer was determined by HPLC using a YMC Amylose SA column (n-hexane:i-PrOH = 3:1, 1 mL/min, 10 °C, retention times: 21.0 and 35.5 min). The enantiomeric excess of the major diastereomer was determined by HPLC using a YMC Cellulose SB column (n-hexane:i-PrOH = 10:1, 1 mL/min, 10 °C, retention times: 22.2 min major, 20.2 min minor).

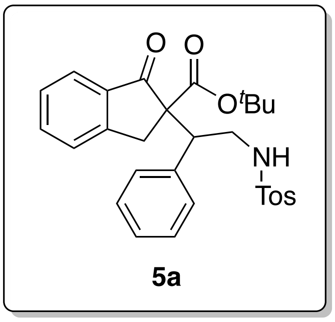

Analytical data for compound **5a** (e.r., d.r., and yield depending on the conditions are given in the main manuscript).

HRMS (ESI): m/z calculated for $C_{29}H_{32}NO_5S^+$: 506.2001 [M+H]$^+$; found: 506.1992

Major diastereomer:

^{1}H-NMR (700 MHz, $CDCl_3$, 298.0 K): δ /ppm = 7.67 (d, J = 8.2 Hz, 2H), 7.49 (d, J = 7.7 Hz, 1H), 7.46 (t, J = 7.2 Hz, 1H), 7.32 (d, J = 7.7 Hz, 1H), 7.28 (d, J = 8.2 Hz, 2H), 7.21 (t, J = 7.2 Hz, 1H), 7.11-7.05 (m, 5H), 4.42-4.40 (m, 1H), 3.87 (dd, J_1 = 9.7 Hz, J_2 = 5.7 Hz, 1H), 3.69 (d, J = 17.2 Hz, 1H), 3.65-3.61 (m, 1H), 3.38-3.34 (m, 1H), 3.22 (d, J = 17.2 Hz, 1H), 2.43 (s, 3H), 1.37 (s, 9H); ^{13}C-NMR (176 MHz, $CDCl_3$, 298.0 K): δ /ppm = 200.7, 169.0, 153.2, 143.6, 137.2, 136.2, 135.2, 135.0, 129.9, 129.6, 128.7, 127.8, 127.6, 127.3, 126.0, 124.7, 83.1, 65.4, 48.0, 44.4, 33.3, 27.8, 21.6.

Minor diastereomer:

^{1}H-NMR (700 MHz, $CDCl_3$, 298.0 K): δ /ppm = 7.73 (d, J = 7.5 Hz, 2H), 7.56 (d, J = 8.1 Hz, 2H), 7.56-7.54 (m, 1H), 7.37-7.34 (m, 2H), 7.22-7.18 (m, 5H), 7.11-7.08 (m, 2H), 4.20-4.18 (m, 1H), 3.90-3.88 (m, 1H), 3.64 (d, J = 16.8 Hz, 1H), 3.40-3.36 (m, 1H), 3.33-3.29 (m, 1H), 3.27 (d, J = 16.8 Hz, 1H), 2.40 (s, 3H), 1.20 (s, 9H); ^{13}C-NMR (176 MHz, $CDCl_3$, 298.0 K): δ /ppm = 202.1, 168.5, 153.2, 143.4, 137.9, 137.0, 135.7, 135.4, 129.8, 129.2, 128.8, 127.9, 127.8, 127.2, 126.3, 124.7, 82.8, 65.2, 49.0, 45.5, 34.7, 27.6, 21.6.

2.1.2. General procedure for the racemic application scope

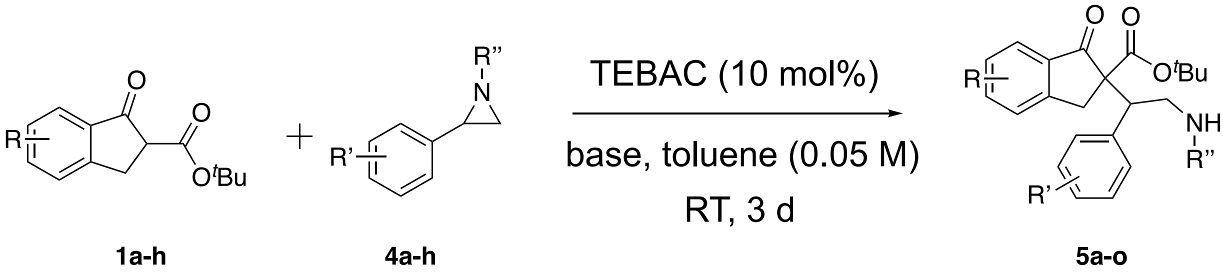

A mixture of 0.1 mmol β-ketoester **1a-h** (1 equiv.), 2.2 mg TEBAC (0.01 mmol, 10 mol%) and 0.2 mmol base (2 equiv.) was dissolved in 5 mL toluene (Ar-atmosphere). Then 0.2 mmol of aziridine **4a-h** (2 equiv.) were added in one portion. After a reaction time of three days the mixture was filtrated through a plug of Na_2SO_4 and washed with DCM. The crude product, obtained after evaporation of the solvent, was subjected to column chromatography (silica gel, heptanes:EtOAc = 5:1) to isolate products **5a-o** and **6** as mixtures of two diastereomers.

The diastereomers were in some cases separated using preparative HPLC (Grace Alltima Silica 10 µm 250x10 mm, n-hexane:EtOAc, 5 mL/min) but otherwise the mixture of diastereomers was analysed and in some cases the signals for the minor diastereomer were hardly detectable (i.e. in the 13C NMR) and mainly assigned with HSQC and HMBC methods.

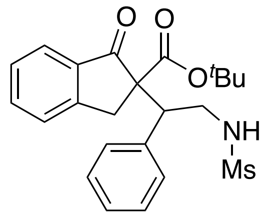

5b

Compound **5b** was prepared according to the general procedure described in 2.1.2 using Cs_2CO_3 as base and was obtained as a white residue in a yield of 4%. Only one diastereomer was detected.

HRMS (ESI): m/z calculated for $C_{23}H_{31}N_2O_5S^+$: 447.1954 $[M+NH_4]^+$; found: 447.1946

^{1}H-NMR (700 MHz, $CDCl_3$, 298.0 K): δ /ppm = 7.72 (d, J = 7.6 Hz, 1H), 7.63 (t, J = 7.8 Hz, 1H), 7.52 (d, J = 7.8 Hz, 1H), 7.41-7.29 (m, 10H), 5.70 (d, J = 7.6 Hz, 1H), 4.70-4.67 (m, 1H), 3.77 (d, J = 7.5 Hz, 1H), 3.27 (d, J = 17.2 Hz, 1H), 2.94 (dd, J_1 = 10.9 Hz, J_2 = 15.0 Hz, 1H), 2.45 (s, 3H), 1.84 (dd, J_1 = 5.3 Hz, J_2 = 15.0 Hz, 1H), 1.39 (s, 9H).

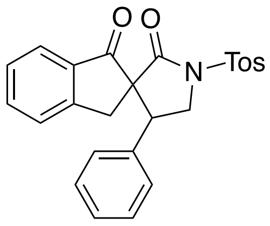

6

Compound **6** was prepared by the general procedure described in 2.1.2 starting from **1c** or **1d** using Cs_2CO_3 or K_3PO_4 (54% yield from **1d** and 17% from **1c**). Only one diastereomer was formed.

HRMS (ESI): m/z calculated for $C_{25}H_{25}N_2O_4S^+$: 449.1535 $[M+NH_4]^+$; found: 449.1528

^{1}H-NMR (300 MHz, $CDCl_3$, 298.0 K): δ /ppm = 8.02 (d, J = 8.3 Hz, 1H), 7.47-7.42 (m, 1H), 7.36 (d, J = 4.1 Hz, 2H), 7.37-7.32 (m, 2H), 7.20-7.13 (m, 6H), 4.68 (dd, J_1 = 11.4 Hz, J_2 = 9.4 Hz, 1H), 4.33 (dd, J_1 = 9.4 Hz, J_2 = 7.8 Hz, 1H), 3.80-3.73 (m, 1H), 3.73 (d, J = 17.5 Hz, 1H), 3.12 (d, J = 17.5 Hz, 1H), 2.45 (s, 3H); ^{13}C-NMR (176 MHz, $CDCl_3$, 298.0 K): δ /ppm = 201.1, 171.0, 153.0, 145.5, 135.7, 135.0, 134.9, 133.5, 129.8, 128.8, 128.5, 128.5, 128.4, 127.9, 126.1, 124.5, 64.9, 49.3, 48.7, 34.8, 21.9.

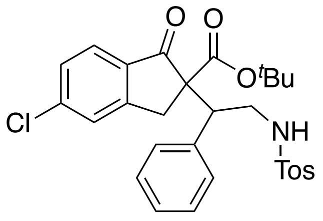

5e

Compound **5e** was prepared by the general procedure described in 2.1.2 using K_3PO_4 as base and was obtained as a colourless residue in a yield of 63% and with a d.r. of 1:4.

HRMS (ESI): m/z calculated for $C_{29}H_{34}ClN_2O_5S^+$: 557.1877 $[M+NH_4]^+$; found: 557.1870

Major diastereomer:

^{1}H-NMR (300 MHz, $CDCl_3$, 298.0 K): δ /ppm = 7.67 (d, J = 8.3 Hz, 2H), 7.41 (d, J = 8.2 Hz, 1H), 7.32-7.27 (m, 3H), 7.20-7.17 (m, 1H), 7.12-7.09 (m, 3H), 7.04-7.01 (m, 2H), 4.39-4.37 (m, 1H), 3.93-3.89 (m, 1H), 3.68 (d, J = 17.3 Hz, 1H), 3.64-3.55 (m, 1H), 3.36-3.28 (m, 1H), 3.20 (d, J = 17.3 Hz, 1H), 2.43 (s, 3H), 1.38 (s, 9H); ^{13}C-NMR (176 MHz, $CDCl_3$, 298.0 K): δ /ppm = 199.4, 168.9, 154.7, 144.1, 143.7, 135.7, 133.4, 129.9, 129.5, 128.8, 128.5, 128.0, 127.3, 126.3, 125.7, 109.4, 83.5, 65.5, 47.8, 44.1, 32.7, 27.8, 21.7.

Minor diastereomer:

^{1}H-NMR (300 MHz, $CDCl_3$, 298.0 K): δ /ppm = 7.67 (d, J = 8.3 Hz, 1H), 7.54 (d, J = 8.2 Hz, 2H), 7.36-7.20 (m, 8H), 7.10-7.07 (m, 1H), 4.15-4.12 (m, 1H), 3.94-3.88 (m, 1H), 3.64 (d, J = 17.1 Hz, 1H), 3.36-3.26 (m, 1H), 3.27 (d, J = 22.2 Hz, 1H), 2.41 (s, 3H), 1.38 (s, 9H).

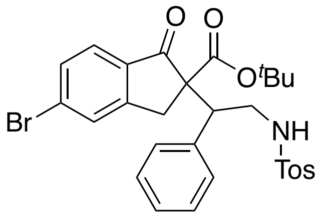

5f

Compound **5f** was prepared by the general procedure described in 2.1.2 using K_3PO_4 as base and was obtained as a white residue in a yield of 79% and with a d.r. of 1:6.

HRMS (ESI): m/z calculated for $C_{29}H_{34}BrN_2O_5S^+$: 601.1372 $[M+NH_4]^+$; found: 601.1363

Major diastereomer:

^{1}H-NMR (300 MHz, $CDCl_3$, 298.0 K): δ /ppm = 7.66 (d, J = 4.2 Hz, 2H), 7.51 (s, 1H), 7.34-7.27 (m, 4H), 7.11-7.08 (m, 3H), 7.03-7.00 (m, 2H), 4.37 (dd, J_1 = 8.5 Hz, J_2 = 3.7 Hz, 1H), 3.90 (dd, J_1 = 9.8 Hz, J_2 = 5.7 Hz, 1H), 3.68 (d, J = 17.5 Hz, 1H), 3.64-3.54 (m, 1H), 3.36-3.28 (m, 1H), 3.20 (d, J = 17.5 Hz, 1H), 2.43 (s, 3H), 1.38 (s, 9H); ^{13}C-NMR (176 MHz, $CDCl_3$, 298.0 K): δ /ppm = 200.5, 168.5, 154.8, 143.8, 143.7, 136.9, 135.7, 133.7, 131.3, 130.8, 129.9, 129.5, 129.3, 128.8, 128.0, 127.3, 125.8, 83.5, 65.5, 47.9, 44.1, 32.7, 27.8, 21.7.

Minor diastereomer:

^{1}H-NMR (300 MHz, $CDCl_3$, 298.0 K): δ /ppm = 7.59 (d, J = 8.2 Hz, 1H), 7.55-7.49 (m, 4H), 7.23-7.20 (m, 5H), 7.10-7.07 (m, 2H), 4.15-4.12 (m, 1H), 3.91 (t, J = 8.1 Hz, 1H), 3.64 (d, J = 17.0 Hz, 1H), 3.37-3.32 (m, 1H), 3.29-3.24 (m, 1H), 3.26 (d, J = 17.0 Hz, 1H), 2.41 (s, 3H), 1.18 (s, 9H); ^{13}C-NMR (176 MHz, $CDCl_3$, 298.0 K): δ /ppm = 200.9, 168.0, 154.8, 143.6, 137.8, 136.7, 134.2, 131.5, 131.0, 129.8, 129.6, 129.1, 128.9, 128.0, 127.1, 125.8, 83.1, 65.4, 48.7, 45.7, 29.8, 27.5, 21.7.

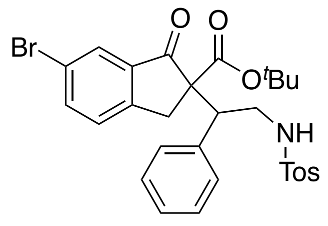

5g

Compound **5g** was prepared by the general procedure described in 2.1.2 using Cs_2CO_3 as base and was obtained as a white solid in a yield of 50% and with a d.r. of 1:3.5.

HRMS (ESI): m/z calculated for $C_{29}H_{34}BrN_2O_5S^+$: 601.1372 $[M+NH_4]^+$; found: 601.1368

Major diastereomer:

^{1}H-NMR (300 MHz, $CDCl_3$, 298.0 K): δ /ppm = 7.67 (d, J = 8.3 Hz, 2H), 7.60-7.55 (m, 2H), 7.30-7.21 (m, 3H), 7.12-7.09 (m, 3H), 7.04-7.01 (m, 2H), 4.40-4.36 (m, 1H), 3.94-3.88 (m, 1H), 3.65-3.54 (m, 1H), 3.60 (d, J = 17.2 Hz, 1H), 3.36-3.28 (m, 1H), 3.15 (d, J = 17.2 Hz, 1H), 2.43 (s, 3H), 1.37 (s, 9H); ^{13}C-NMR (176 MHz, $CDCl_3$, 298.0 K): δ /ppm = 199.3, 168.5, 151.8, 143.7, 138.0, 136.9, 136.7, 135.7, 129.9, 129.5, 128.8, 128.0, 127.5, 127.4, 127.2, 121.7, 83.5, 65.8, 47.7, 44.2, 32.7, 27.8, 21.7.

Minor diastereomer:

^{1}H-NMR (300 MHz, $CDCl_3$, 298.0 K): δ /ppm = 7.85-7.53 (m, 3H), 7.28-7.20 (m, 8H), 7.10-7.07 (m, 1H), 4.17-4.11 (m, 1H), 3.93-3.82 (m, 1H), 3.61 (d, J = 17.0 Hz, 1H), 3.39-3.23 (m, 1H), 3.25 (d, J = 17.0 Hz, 1H), 2.41 (s, 3H), 1.18 (s, 9H); ^{13}C-NMR (176 MHz, $CDCl_3$, 298.0 K): δ /ppm = 200.8, 168.0, 151.8, 144.1, 143.6, 138.2, 137.2, 136.7, 129.8, 129.1, 128.9, 128.0, 127.8, 127.5, 127.2, 121.9, 83.1, 65.7, 48.9, 31.7, 27.6, 22.8, 14.3.

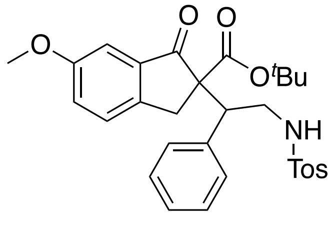

5h

Compound **5h** was prepared by the general procedure described in 2.1.2 using Cs_2CO_3 as base and was obtained as a white solid in a yield of 74% and with a d.r. of 1:4.5.

HRMS (ESI): m/z calculated for $C_{30}H_{37}N_2O_6S^+$: 553.2372 $[M+NH_4]^+$; found: 553.2364

Major diastereomer:

^{1}H-NMR (300 MHz, $CDCl_3$, 298.0 K): δ /ppm = 7.67 (d, J = 8.3 Hz, 2H), 7.30-7.20 (m, 3H), 7.11-7.04 (m, 6H), 6.92-6.91 (m, 1H), 4.42-4.38 (m, 1H), 3.92-3.87 (m, 1H), 3.73 (s, 3H), 3.65-3.56 (m, 1H), 3.60 (d, J = 17.1 Hz, 1H), 3.37-3.28 (m, 1H), 3.15 (d, J = 17.1 Hz, 1H), 2.43 (s, 3H), 1.37 (s, 9H); ^{13}C-NMR (176 MHz, $CDCl_3$, 298.0 K): δ /ppm = 200.6, 169.0, 159.5, 146.1, 143.5, 137.1, 136.2, 136.1, 129.8, 129.6, 128.7, 127.8, 127.3, 126.7, 124.7, 105.7, 83.1, 66.1, 55.6, 48.0, 44.4, 32.7, 27.8, 21.6.

Minor diastereomer:

^{1}H-NMR (300 MHz, $CDCl_3$, 298.0 K): δ /ppm = 7.60-7.55 (m, 3H), 7.24-7.14 (m, 9H), 4.22-4.17 (m, 1H), 3.92-3.84 (m, 1H), 3.83 (s, 3H), 3.74 (d, J = 17.7 Hz, 1H), 3.55 (d, J = 16.9 Hz, 1H), 3.38-3.30 (m, 1H), 3.17 (d, J = 16.9 Hz, 1H), 2.41 (s, 3H), 1.21 (s, 9H); ^{13}C-NMR (176 MHz, $CDCl_3$, 298.0 K): δ /ppm = 201.9, 168.5, 159.7, 146.1, 143.3, 137.8, 137.0, 136.8, 129.7, 129.2, 128.8, 127.8, 127.2, 126.9, 124.8, 105.8, 82.7, 65.8, 55.7, 49.0, 45.4, 34.0, 27.6, 21.6.

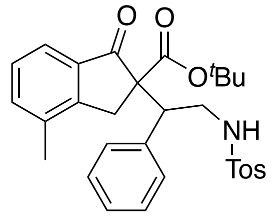

5i

Compound **5i** was prepared by the general procedure described in 2.1.2 using K_3PO_4 as base and was obtained as a colourless residue in a yield of 39% and with a d.r. of 1:5.

HRMS (ESI): m/z calculated for $C_{30}H_{37}N_2O_5S^+$: 537.2423 $[M+NH_4]^+$; found: 537.2415

Major diastereomer:

^{1}H-NMR (300 MHz, $CDCl_3$, 298.0 K): δ /ppm = 7.68 (d, J = 8.3 Hz, 2H), 7.34-7.30 (m, 4H), 7.13 (d, J = 7.5 Hz, 1H), 7.09-7.04 (m, 5H), 4.42-4.38 (m, 1H), 3.92-3.87 (m, 1H), 3.66-3.57 (m, 1H), 3.56 (d, J = 17.4 Hz, 1H), 3.43-3.34 (m, 1H), 3.04 (d, J = 17.4 Hz, 1H), 2.43 (s, 3H), 2.28 (s, 3H), 1.37 (s, 9H); ^{13}C-NMR (176 MHz, $CDCl_3$, 298.0 K): δ /ppm = 200.8, 168.9, 152.0, 143.5, 137.2, 136.1, 135.6, 135.1, 134.7, 129.8, 129.5, 128.6, 127.8, 127.7, 127.3, 122.0, 83.1, 65.4, 48.1, 44.2, 32.1, 27.8, 21.6, 17.8

Minor diastereomer:

^{1}H-NMR (300 MHz, $CDCl_3$, 298.0 K): δ /ppm = 7.61-7.53 (m, 3H), 7.39-7.30 (m, 2H), 7.22-7.20 (m, 5H), 7.13-7.10 (m, 2H), 4.20-4.14 (m, 1H), 3.93 (t, J = 8.3 Hz, 1H), 3.59 (d, J = 17.1 Hz, 1H), 3.34-3.27 (m, 1H), 3.11 (d, J = 17.1 Hz, 1H), 2.41 (s, 3H), 2.30 (s, 3H), 1.19 (s, 9H); ^{13}C-NMR (176 MHz, $CDCl_3$, 298.0 K): δ /ppm = 202.1, 168.4, 152.2, 143.3, 138.0,

137.0, 135.9, 135.5, 135.3, 129.7, 129.1, 128.7, 127.9, 127.8, 127.2, 122.0, 82.7, 65.3, 48.9, 45.6, 33.2, 27.6, 21.6, 17.8

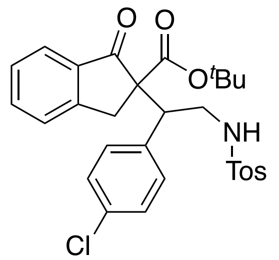

5j

Compound **5j** was prepared by the general procedure described in 2.3.2 using Cs_2CO_3 as base and was obtained as a white residue in a yield of 80% and with a d.r. of 1:4.

HRMS (ESI): m/z calculated for $C_{29}H_{34}ClN_2O_5S^+$: 557.1877 $[M+NH_4]^+$; found: 557.1883

Major diastereomer:

^{1}H-NMR (300 MHz, $CDCl_3$, 298.0 K): δ /ppm = 7.65 (d, J = 8.3 Hz, 2H), 7.52-7.48 (m, 2H), 7.36-7.22 (m, 4H), 7.07-6.98 (m, 4H), 4.41-4.37 (m, 1H), 3.90-3.85 (m, 1H), 3.69 (d, J = 17.2 Hz, 1H), 3.62-3.53 (m, 1H), 3.35-3.26 (m, 1H), 3.18 (d, J = 17.2 Hz, 1H), 2.43 (s, 3H), 1.35 (s, 9H); ^{13}C-NMR (176 MHz, $CDCl_3$, 298.0 K): δ /ppm = 200.6, 168.8, 153.0, 143.7, 136.9, 135.5, 134.8, 133.8, 130.9, 129.9, 128.8, 127.8, 127.2, 126.0, 125.0, 124.8, 83.3, 65.1, 47.3, 44.4, 33.2, 27.8, 21.7

Minor diastereomer:

^{1}H-NMR (300 MHz, $CDCl_3$, 298.0 K): δ /ppm = 7.73 (d, J = 7.3 Hz, 1H), 7.58 (d, J = 9.2 Hz, 1H), 7.53 (d, J = 8.3 Hz, 2H), 7.37 (d, J = 7.1 Hz, 2H), 7.20 (d, J = 8.1 Hz, 2H), 7.14 (d, J = 8.5 Hz, 2H), 7.00 (d, J = 8.5 Hz, 2H), 4.23-4.19 (m, 1H), 3.88-3.83 (m, 1H), 3.60 (d, J = 16.7 Hz, 1H), 3.35-3.27 (m, 2H), 3.20 (d, J = 16.7 Hz, 1H), 2.40 (s, 3H), 1.21 (s, 9H); ^{13}C-NMR (176 MHz, $CDCl_3$, 298.0 K): δ /ppm = 201.8, 168.4, 153.0, 143.5, 136.7, 136.4, 135.6, 133.7, 130.5, 129.8, 129.8, 128.9, 127.9, 127.3, 127.1, 126.3, 124.7, 83.0, 64.9, 48.5, 45.4, 34.6, 27.5, 21.7

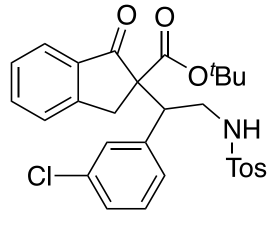

5k

Compound **5k** was prepared by the general procedure described in 2.1.2 using Cs_2CO_3 as the base and was obtained as a white residue in a yield of 37% and with a d.r. of 2:1.

HRMS (ESI): m/z calculated for $C_{29}H_{34}ClN_2O_5S^+$: 557.1877 $[M+NH_4]^+$; found: 557.1869

Major diastereomer:

^{1}H-NMR (700 MHz, $CDCl_3$, 298.0 K): δ /ppm = 7.66 (d, J = 8.2 Hz, 2H), 7.53-7.49 (m, 2H), 7.36 (d, J = 7.6 Hz, 1H), 7.28-7.23 (m, 3H), 7.07-7.03 (m, 2H), 6.99-6.97 (m, 2H), 4.45-4.43 (m, 1H), 3.81 (dd, J_1 = 9.5 Hz, J_2 = 5.7 Hz, 1H), 3.69 (d, J = 17.2 Hz, 1H), 3.65-3.62 (m, 1H), 3.35-3.31 (m, 1H), 3.18 (d, J = 17.2 Hz, 1H), 2.43 (s, 3H), 1.37 (s, 9H); ^{13}C-NMR (176 MHz,

$CDCl_3$, 298.0 K): δ /ppm = 200.4, 168.8, 152.9, 143.7, 138.6, 137.1, 135.4, 134.9, 134.5, 129.9, 129.5, 128.0, 127.9, 127.2, 126.0, 124.7, 83.3, 65.0, 47.9, 44.4, 33.7, 27.8, 21.6.

Minor diastereomer:

^{1}H-NMR (300 MHz, $CDCl_3$, 298.0 K): δ /ppm = 7.75 (d, J = 8.0 Hz, 1H), 7.61-7.54 (m, 3H), 7.41-7.37 (m, 2H), 7.22-7.14 (m, 5H), 7.02-7.00 (m, 2H), 4.23-4.19 (m, 1H), 3.89-3.84 (m, 1H), 3.66 (d, J = 16.8 Hz, 1H), 3.37-3.26 (m, 2H), 3.23 (d, J = 16.8 Hz, 1H), 2.41 (s, 3H), 1.21 (s, 9H); ^{13}C-NMR (176 MHz, $CDCl_3$, 298.0 K): δ /ppm = 201.2, 168.0, 143.5, 140.1, 135.6, 135.4, 130.0, 129.8, 129.8, 129.7, 128.0, 127.9, 127.3, 127.2, 127.1, 127.0, 126.3, 124.7, 83.0, 65.1, 48.6, 45.3, 34.3, 27.5, 21.6.

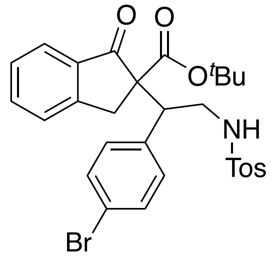

5l

Compound **5l** was prepared by the general procedure described in 2.1.2 using Cs_2CO_3 as base and was obtained as a white residue in a yield of 96% and with a d.r. of 1:3.

HRMS (ESI): m/z calculated for $C_{29}H_{34}BrN_2O_5S^+$: 601.1372 $[M+NH_4]^+$; found: 601.1360

Major diastereomer:

^{1}H-NMR (300 MHz, $CDCl_3$, 298.0 K): δ /ppm = 7.64 (d, J = 8.2 Hz, 2H), 7.53-7.48 (m, 2H), 7.40-7.28 (m, 4H), 7.21 (d, J = 8.5 Hz, 2H), 6.94 (d, J = 8.5 Hz, 2H), 4.41-4.37 (m, 1H), 3.89-3.84 (m, 1H), 3.69 (d, J = 17.3 Hz, 1H), 3.57-3.52 (m, 1H), 3.34-3.29 (m, 1H), 3.17 (d, J = 17.3 Hz, 1H), 2.43 (s, 3H), 1.35 (s, 9H); ^{13}C-NMR (176 MHz, $CDCl_3$, 298.0 K): δ /ppm = 200.7, 168.8, 143.7, 136.9, 135.3, 134.8, 131.8, 131.2, 129.9, 127.8, 127.2, 126.4, 126.0, 124.8, 121.9, 83.3, 65.1, 47.4, 44.4, 33.3, 27.8, 21.7.

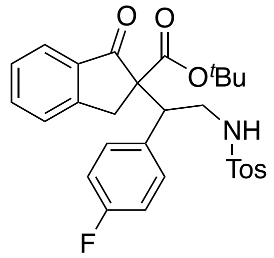

5m

Compound **5m** was prepared by the general procedure described in 2.1.2 using K_3PO_4 as base and was obtained as a white residue in a yield of 75% and with a d.r. of 1:2.5.

HRMS (ESI): m/z calculated for $C_{29}H_{34}FN_2O_5S^+$: 541.2172 $[M+NH_4]^+$; found: 541.2162

Major diastereomer:

^{1}H-NMR (300 MHz, $CDCl_3$, 298.0 K): δ /ppm = 7.67 (d, J = 8.3 Hz, 2H), 7.52-7.47 (m, 2H), 7.36-7.21 (m, 4H), 7.06-7.01 (m, 2H), 6.83-6.75 (m, 2H), 4.41-4.37 (m, 1H), 3.93-3.88 (m, 1H), 3.70 (d, J = 17.3 Hz, 1H), 3.62-3.51 (m, 1H), 3.35-3.26 (m, 1H), 3.19 (d, J = 17.3 Hz, 1H), 2.43 (s, 3H), 1.36 (s, 9H); ^{13}C-NMR (176 MHz, $CDCl_3$, 298.0 K): δ /ppm = 201.2, 168.9, 144.0, 143.6, 137.1, 135.3, 132.0, 131.2, 131.1, 129.8, 129.5, 127.7, 127.2, 126.7, 126.0, 124.7, 115.6, 83.2, 65.2, 47.3, 44.5, 33.3, 28.0, 21.6.

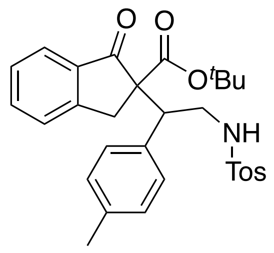

5n

Compound **5n** was prepared by the general procedure described in 2.1.2 using Cs_2CO_3 as base and was obtained as a colourless residue in a yield of 89% and with a d.r. of 1:3.

HRMS (ESI): m/z calculated for $C_{30}H_{37}N_2O_5S^+$: 537.2423 $[M+NH_4]^+$; found: 537.2419

Major diastereomer:

^{1}H-NMR (300 MHz, $CDCl_3$, 298.0 K): δ /ppm = 7.67 (d, J = 8.3 Hz, 2H), 7.52-7.45 (m, 2H), 7.35-7.19 (m, 4H), 6.95-6.88 (m, 4H), 4.39-4.36 (m, 1H), 3.89-3.84 (m, 1H), 3.68 (d, J = 17.3 Hz, 1H), 3.64-3.54 (m, 1H), 3.35-3.26 (m, 1H), 3.21 (d, J = 17.3 Hz, 1H), 2.43 (s, 3H), 2.18 (s, 3H), 1.37 (s, 9H); ^{13}C-NMR (176 MHz, $CDCl_3$, 298.0 K): δ /ppm = 200.6, 168.9, 153.2, 143.5, 137.5, 137.2, 135.1, 135.0, 132.9, 129.8, 129.4, 129.4, 127.5, 127.3, 126.0, 124.6, 83.0, 65.5, 47.5, 44.3, 33.2, 27.8, 21.0, 14.2.

Minor diastereomer:

^{1}H-NMR (300 MHz, $CDCl_3$, 298.0 K): δ /ppm = 7.74-7.65 (m, 2H), 7.59-7.45 (m, 3H), 7.36 (d, J = 8.0 Hz, 1H), 7.22 (d, J = 7.9 Hz, 2H), 6.97 (d, J = 4.1 Hz, 2H), 6.91 (d, J = 3.8 Hz, 2H), 4.39-4.35 (m, 1H), 3.88-3.80 (m, 1H), 3.64 (t, J = 17.8 Hz, 1H), 3.36-3.18 (m, 3H), 2.41 (d, J = 7.8 Hz, 3H), 2.21 (d, J = 24.5 Hz, 3H), 1.28 (d, J = 45.1 Hz, 9H).

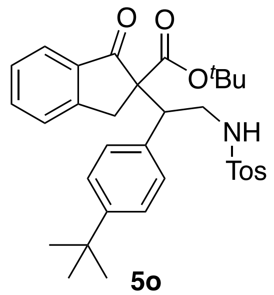

5o

Compound **5o** was prepared by the general procedure described in 2.3.2 using K_3PO_4 as base and was obtained as a white residue in a yield of 90%. Only one diastereomer was formed.

HRMS (ESI): m/z calculated for $C_{33}H_{43}N_2O_5S^+$: 579.2893 $[M+NH_4]^+$; found: 579.2887

^{1}H-NMR (300 MHz, $CDCl_3$, 298.0 K): δ /ppm = 7.67 (d, J = 8.2 Hz, 2H), 7.49-7.43 (m, 2H), 7.33-7.16 (m, 4H), 7.08 (d, J = 8.4 Hz, 2H), 6.95 (d, J = 8.4 Hz, 2H), 4.41-4.37 (m, 1H), 3.85-3.80 (m, 1H), 3.67 (d, J = 17.5 Hz, 1H), 3.62-3.55 (m, 1H), 3.37-3.28 (m, 1H), 3.20 (d, J = 17.5 Hz, 1H), 2.42 (s, 3H), 1.36 (s, 9H), 1.17 (s, 9H); ^{13}C-NMR (176 MHz, $CDCl_3$, 298.0 K): δ /ppm = 200.7, 169.0, 153.2, 150.7, 143.5, 137.2, 135.0, 132.8, 129.8, 129.2, 127.9, 127.4, 127.3, 125.9, 125.5, 124.6, 83.0, 65.5, 47.6, 44.3, 34.4, 31.4, 31.2, 27.8, 21.6.

3. Illustrative NMR Spectra of New Compounds

NOTE: Diastereomers had to be separated by semiprep. HPLC and the minor diastereomers could only be obtained in rather small and diluted amounts (if possible at all) sometimes containing detectable amounts of the major diastereomer.

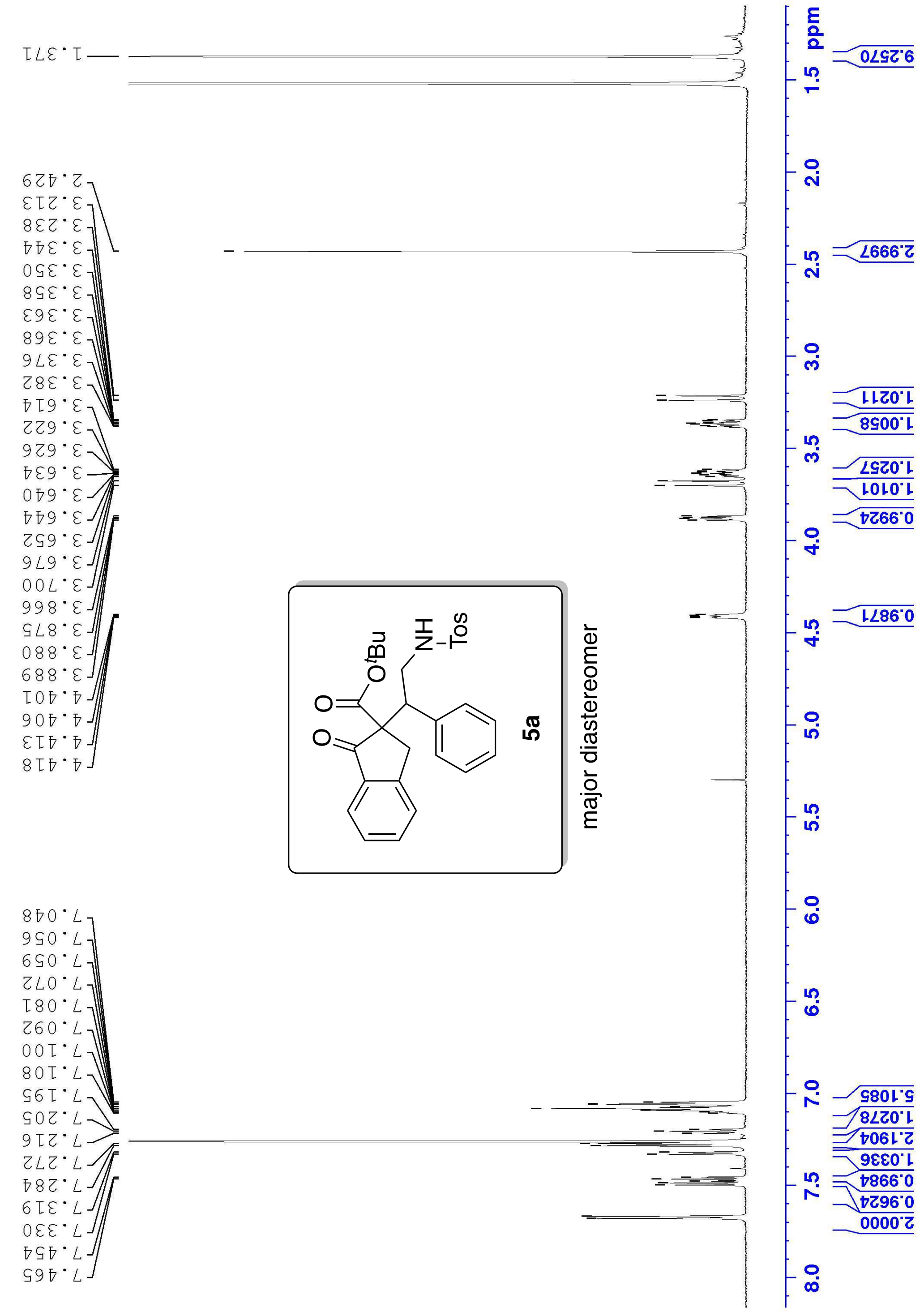

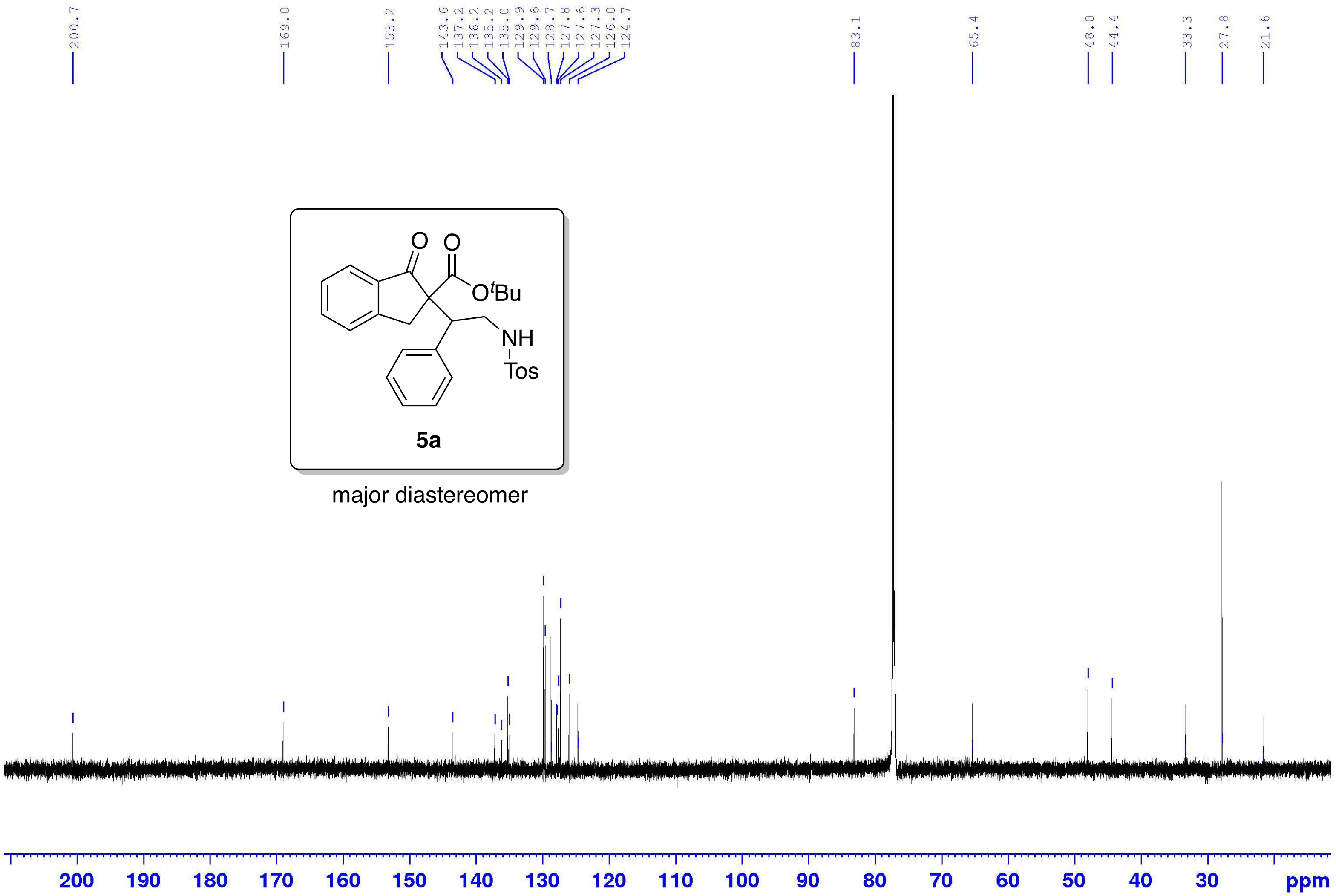
200.7
169.0
153.2
143.6
137.2
136.2
135.2
135.0
129.9
129.6
128.7
127.8
127.6
127.3
126.0
124.7
83.1
65.4
48.0
44.4
33.3
27.8
21.6
O
O
O^tBu
NH
Tos
5a
major diastereomer
200
190
180
170
160
150
140
130
120
110
100
90
80
70
60
50
40
30
ppm

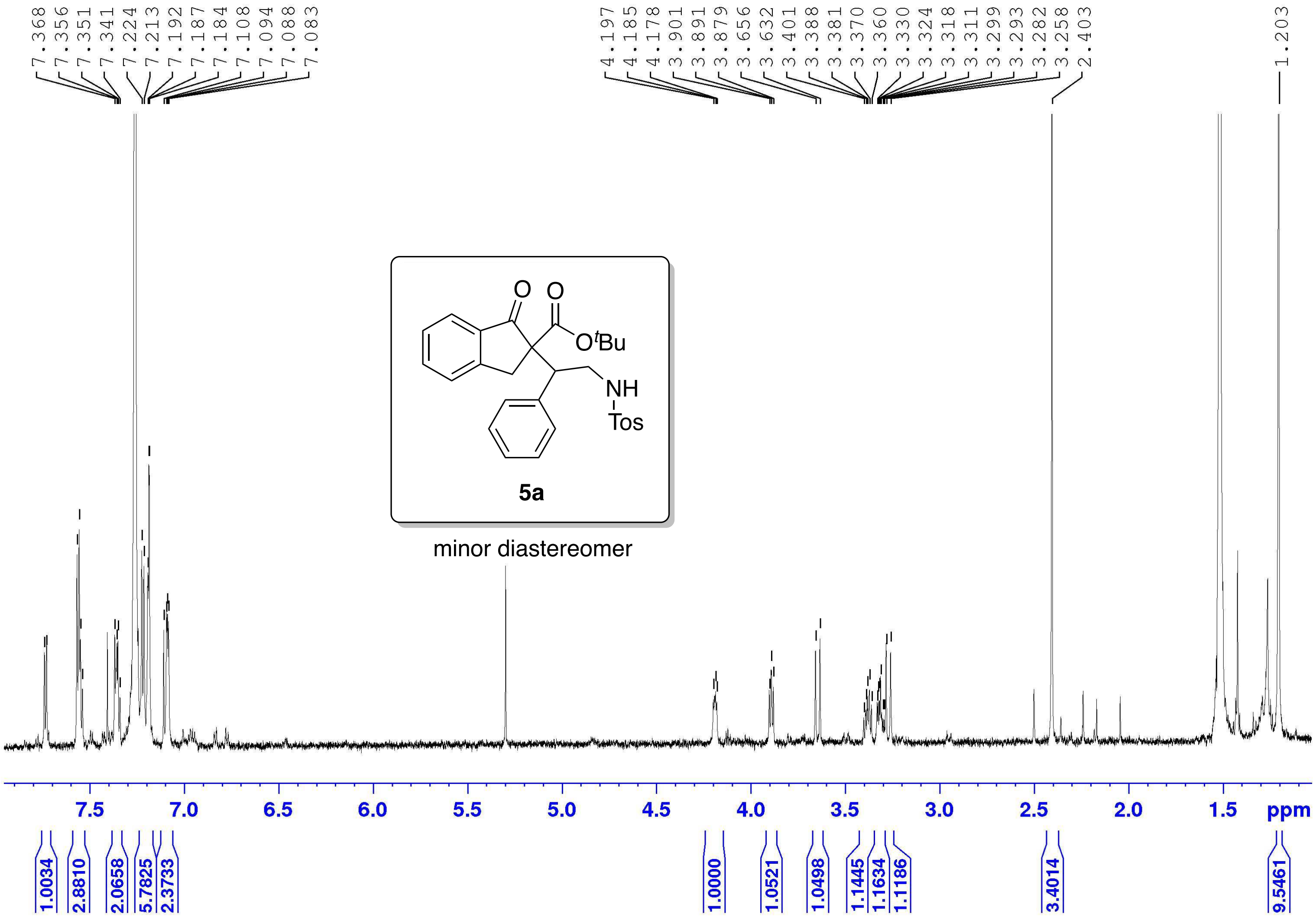
7.368 7.356 7.351 7.341 7.224 7.213 7.192 7.187 7.184 7.108 7.094 7.088 7.083
4.197 4.185 4.178 3.901 3.891 3.879 3.656 3.632 3.401 3.388 3.381 3.370 3.360 3.330 3.324 3.318 3.311 3.299 3.293 3.282 3.258 2.403
1.203
O
O
O^{t}Bu
NH
Tos
5a
minor diastereomer
7.5 7.0 6.5 6.0 5.5 5.0 4.5 4.0 3.5 3.0 2.5 2.0 1.5 ppm
1.0034 2.8810 2.0658 5.7825 2.3733 1.0000 1.0521 1.0498 1.1445 1.1634 1.1186 3.4014 9.5461

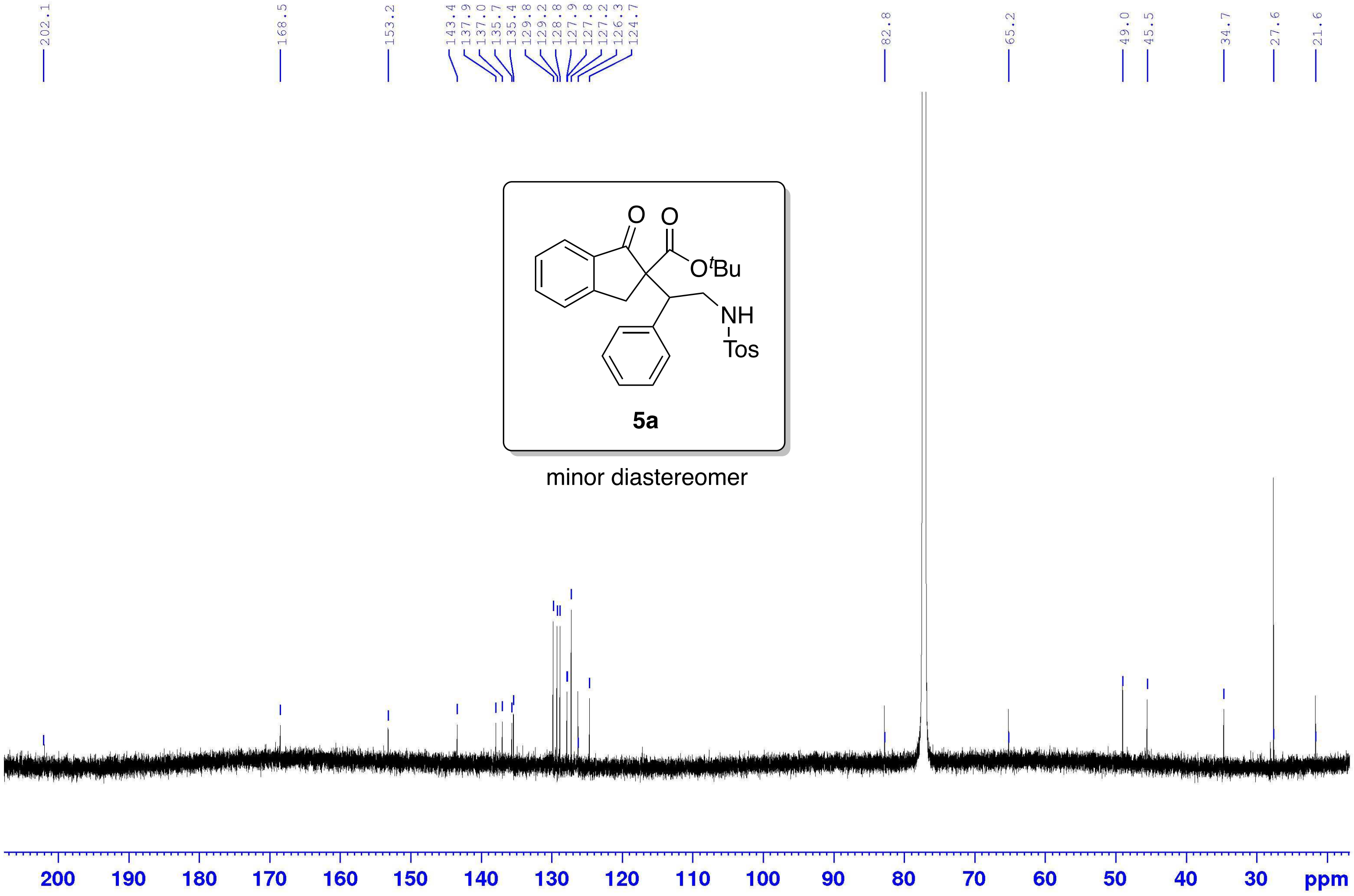
202.1
168.5
153.2
143.4
137.9
137.0
135.7
135.4
129.8
129.2
128.8
127.9
127.8
127.2
126.3
124.7
82.8
65.2
49.0
45.5
34.7
27.6
21.6
O
O
O^tBu
NH
Tos
5a
minor diastereomer
200
190
180
170
160
150
140
130
120
110
100
90
80
70
60
50
40
30
ppm

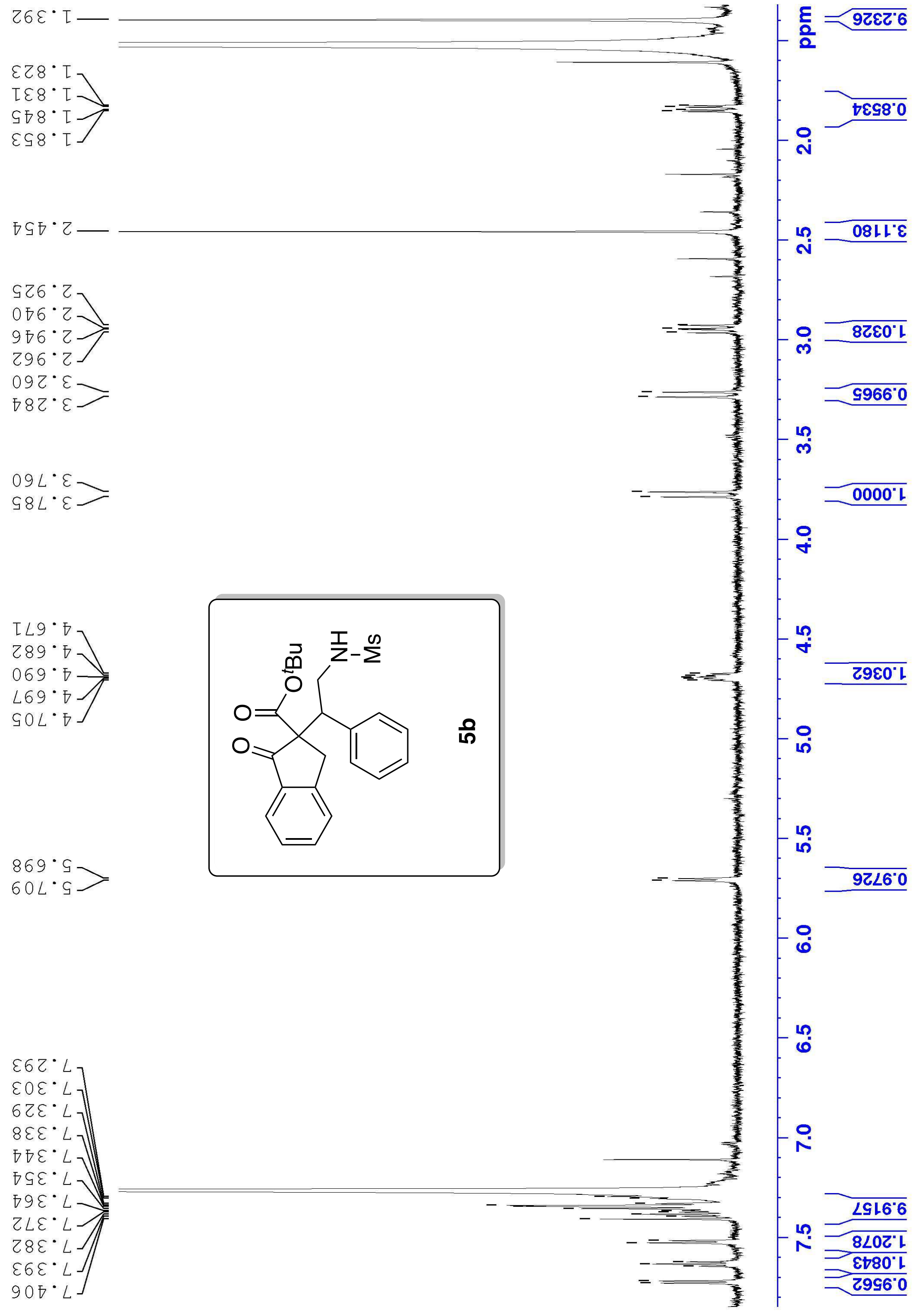
1.392
1.823
1.831
1.845
1.853
2.454
2.925
2.940
2.946
2.962
3.260
3.284
3.760
3.785
4.671
4.682
4.690
4.697
4.705
5.698
5.709
7.293
7.303
7.329
7.338
7.344
7.354
7.364
7.372
7.382
7.393
7.406
ppm
9.2326
0.8534
3.1180
1.0328
0.9965
1.0000
1.0362
0.9726
9.9157
1.2078
1.0843
0.9562
O
O
O^tBu
NH
Ms
5b

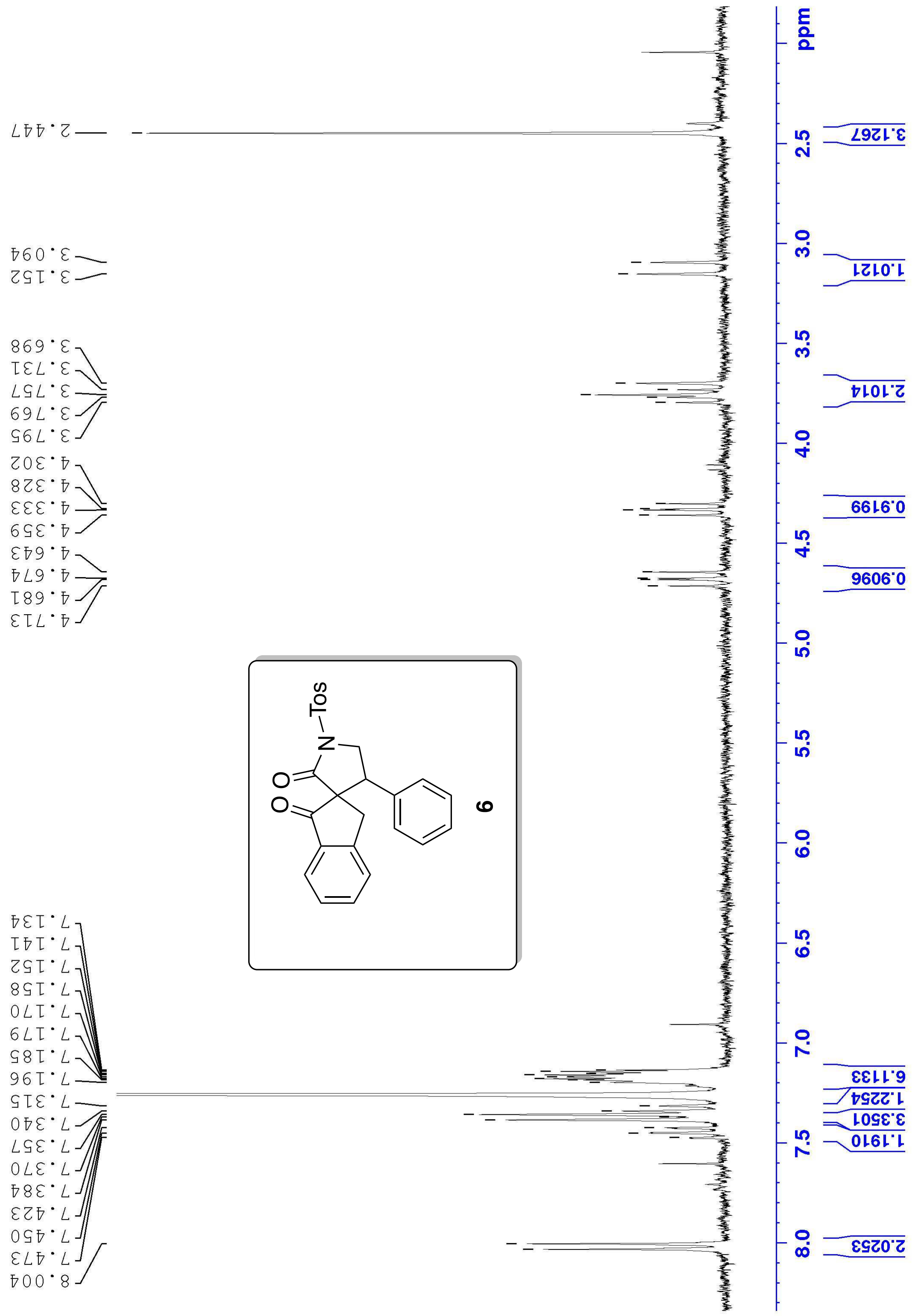
2.447
3.094
3.152
3.698
3.731
3.757
3.769
3.795
4.302
4.328
4.333
4.359
4.643
4.674
4.681
4.713
7.134
7.141
7.152
7.158
7.170
7.179
7.185
7.196
7.315
7.340
7.357
7.370
7.384
7.423
7.450
7.473
8.004
O
O
N
Tos
6
ppm
2.5
3.0
3.5
4.0
4.5
5.0
5.5
6.0
6.5
7.0
7.5
8.0
3.1267
1.0121
2.1014
0.9199
0.9096
6.1133
1.2254
3.3501
1.1910
2.0253

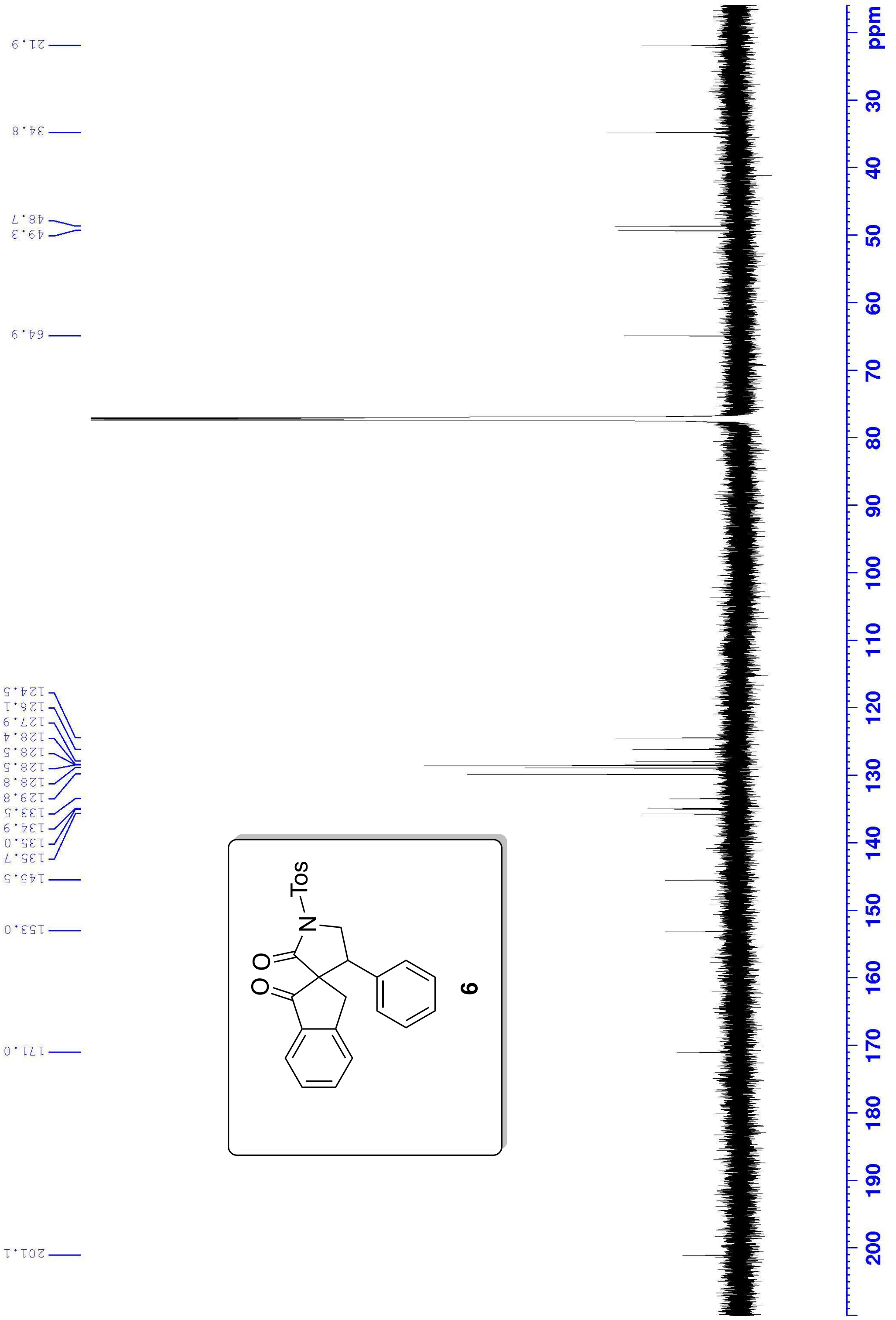

21.9
34.8
48.7
49.3
64.9
124.5
126.1
127.9
128.4
128.5
128.5
128.8
129.8
133.5
134.9
135.0
135.7
145.5
153.0
171.0
201.1
O
O
N
Tos
6
ppm
30
40
50
60
70
80
90
100
110
120
130
140
150
160
170
180
190
200

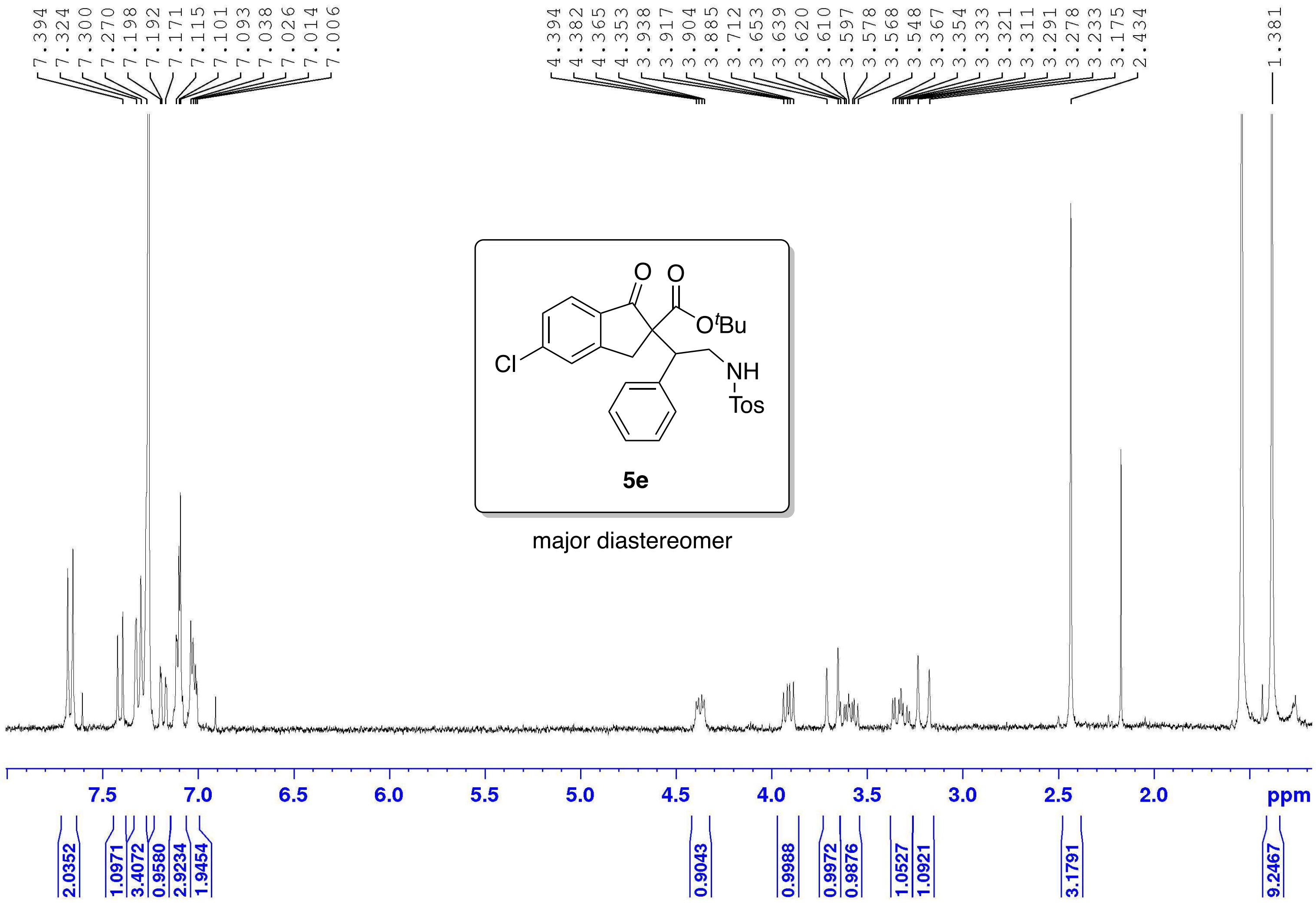
7.394 7.324 7.300 7.270 7.198 7.192 7.171 7.115 7.101 7.093 7.038 7.026 7.014 7.006
4.394 4.382 4.365 4.353 3.938 3.917 3.904 3.885 3.712 3.653 3.639 3.620 3.610 3.597 3.578 3.568 3.548 3.367 3.354 3.333 3.321 3.311 3.291 3.278 3.233 3.175 2.434
1.381
O O
O^tBu
Cl
NH
Tos
5e
major diastereomer
7.5 7.0 6.5 6.0 5.5 5.0 4.5 4.0 3.5 3.0 2.5 2.0 ppm
2.0352 1.0971 3.4072 0.9580 2.9234 1.9454 0.9043 0.9988 0.9972 0.9876 1.0527 1.0921 3.1791 9.2467

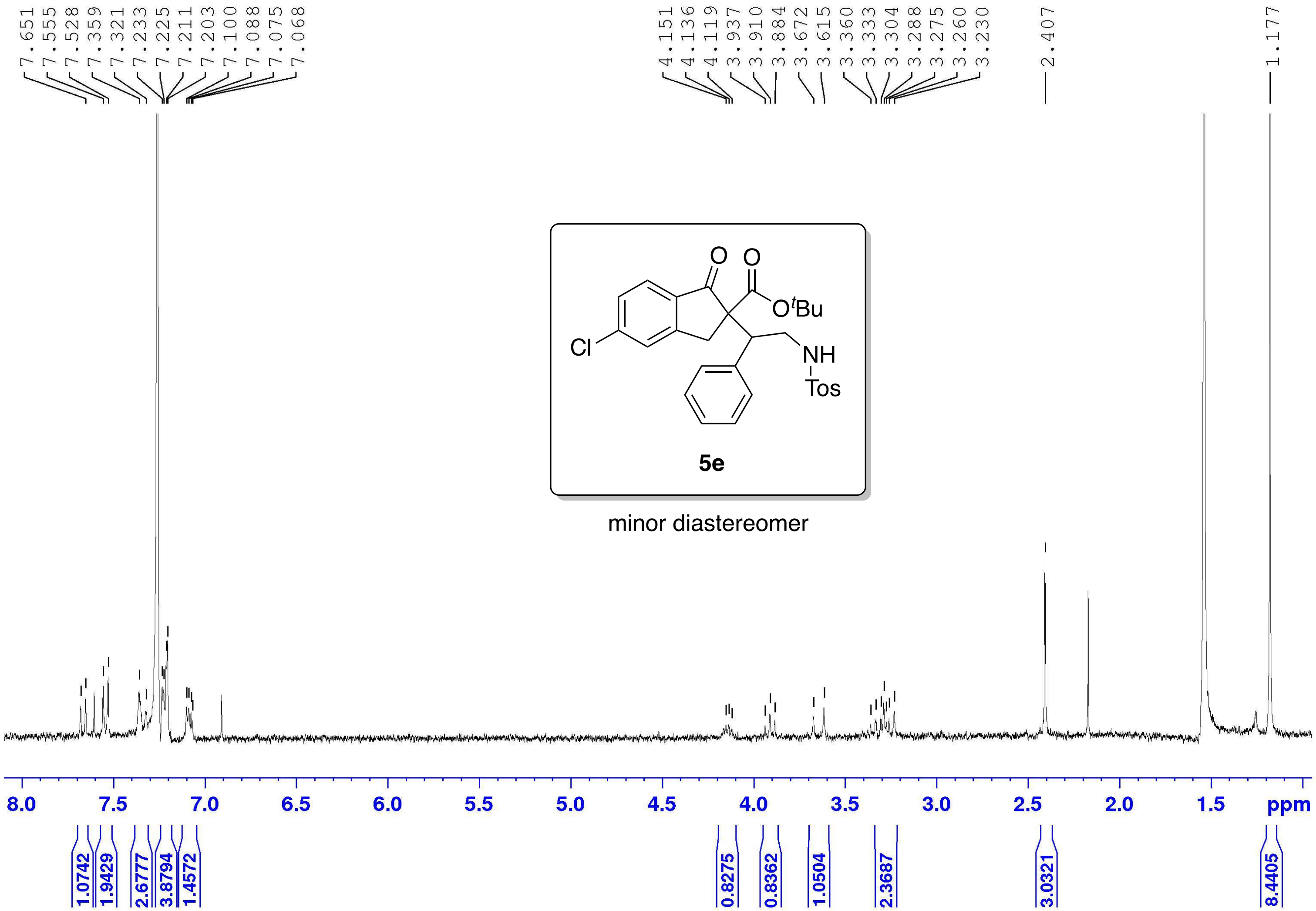
7.651
7.555
7.528
7.359
7.321
7.233
7.225
7.211
7.203
7.100
7.088
7.075
7.068
4.151
4.136
4.119
3.937
3.910
3.884
3.672
3.615
3.360
3.333
3.304
3.288
3.275
3.260
3.230
2.407
1.177
O
O
O^{t}Bu
Cl
NH
Tos
5e
minor diastereomer
8.0
7.5
7.0
6.5
6.0
5.5
5.0
4.5
4.0
3.5
3.0
2.5
2.0
1.5
ppm
1.0742
1.9429
2.6777
3.8794
1.4572
0.8275
0.8362
1.0504
2.3687
3.0321
8.4405

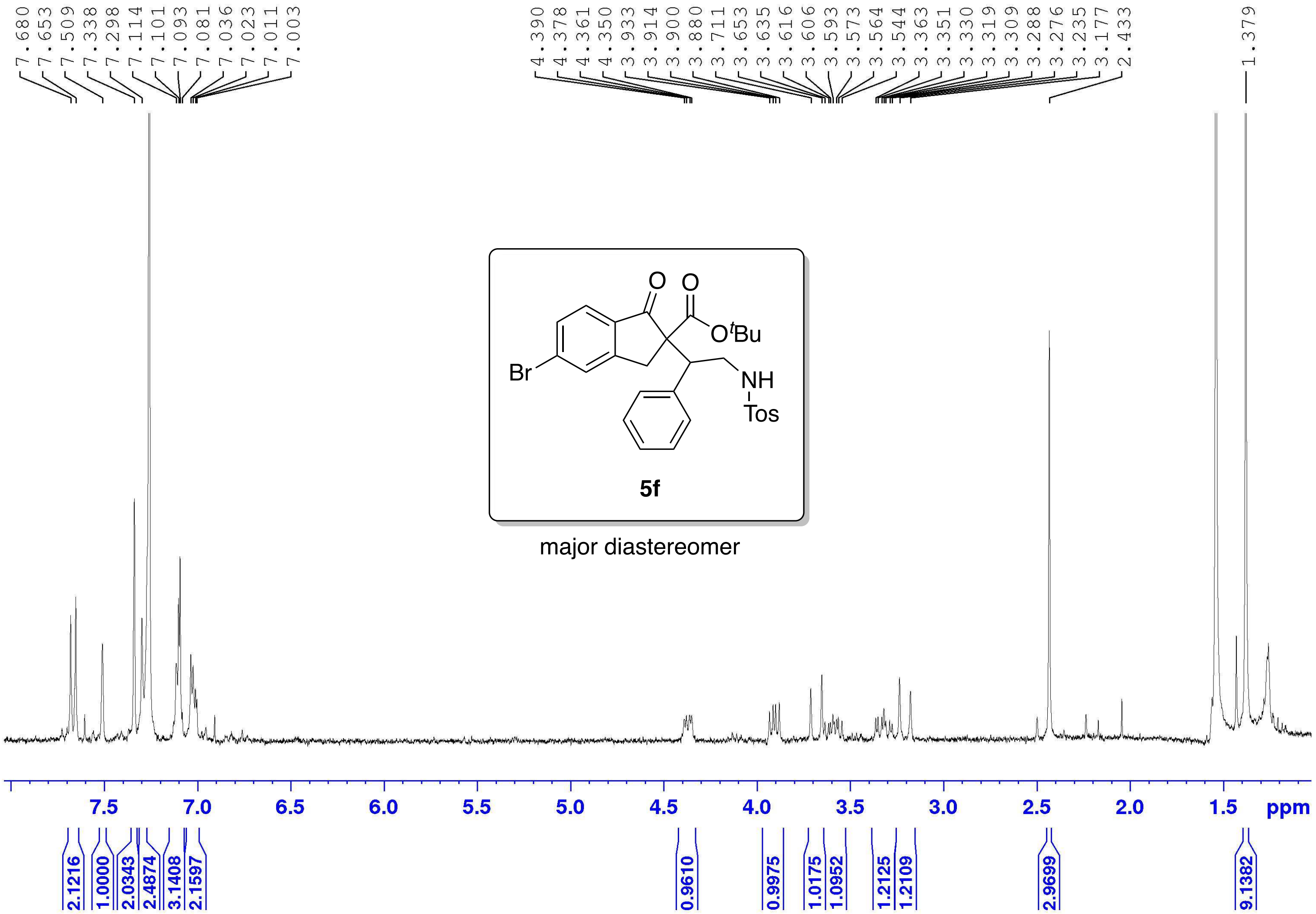

7.680
7.653
7.509
7.338
7.298
7.114
7.101
7.093
7.081
7.036
7.023
7.011
7.003
4.390
4.378
4.361
4.350
3.933
3.914
3.900
3.880
3.711
3.653
3.635
3.616
3.606
3.593
3.573
3.564
3.544
3.363
3.351
3.330
3.319
3.309
3.288
3.276
3.235
3.177
2.433
1.379
O
O
O^{t}Bu
Br
NH
Tos
5f
major diastereomer
7.5
7.0
6.5
6.0
5.5
5.0
4.5
4.0
3.5
3.0
2.5
2.0
1.5
ppm
2.1216
1.0000
2.0343
2.4874
3.1408
2.1597
0.9610
0.9975
1.0175
1.0952
1.2125
1.2109
2.9699
9.1382

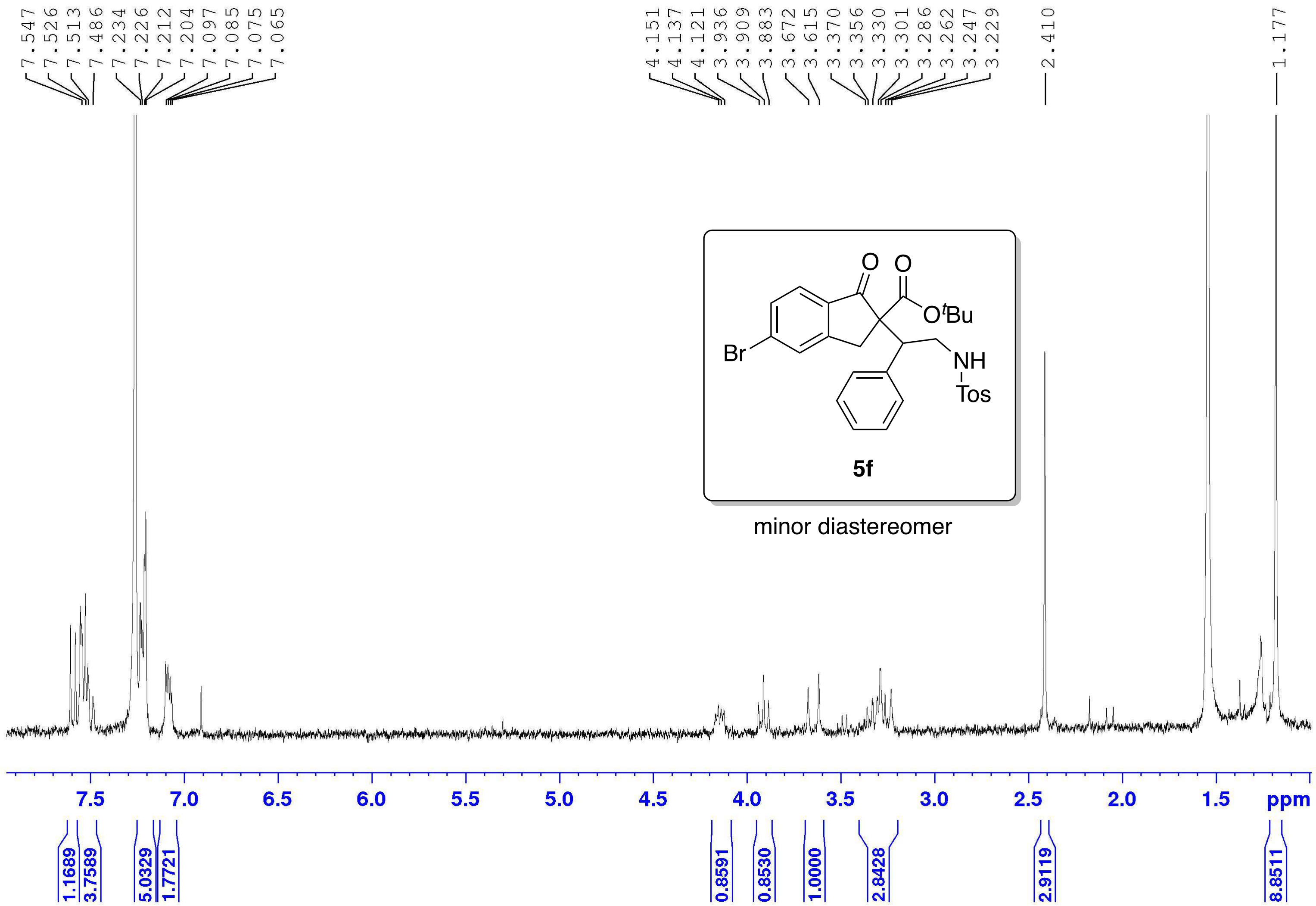
7.547
7.526
7.513
7.486
7.234
7.226
7.212
7.204
7.097
7.085
7.075
7.065
4.151
4.137
4.121
3.936
3.909
3.883
3.672
3.615
3.370
3.356
3.330
3.301
3.286
3.262
3.247
3.229
2.410
1.177
Br
O
O
O^{t}Bu
NH
Tos
5f
minor diastereomer
7.5
7.0
6.5
6.0
5.5
5.0
4.5
4.0
3.5
3.0
2.5
2.0
1.5
ppm
1.1689
3.7589
5.0329
1.7721
0.8591
0.8530
1.0000
2.8428
2.9119
8.8511

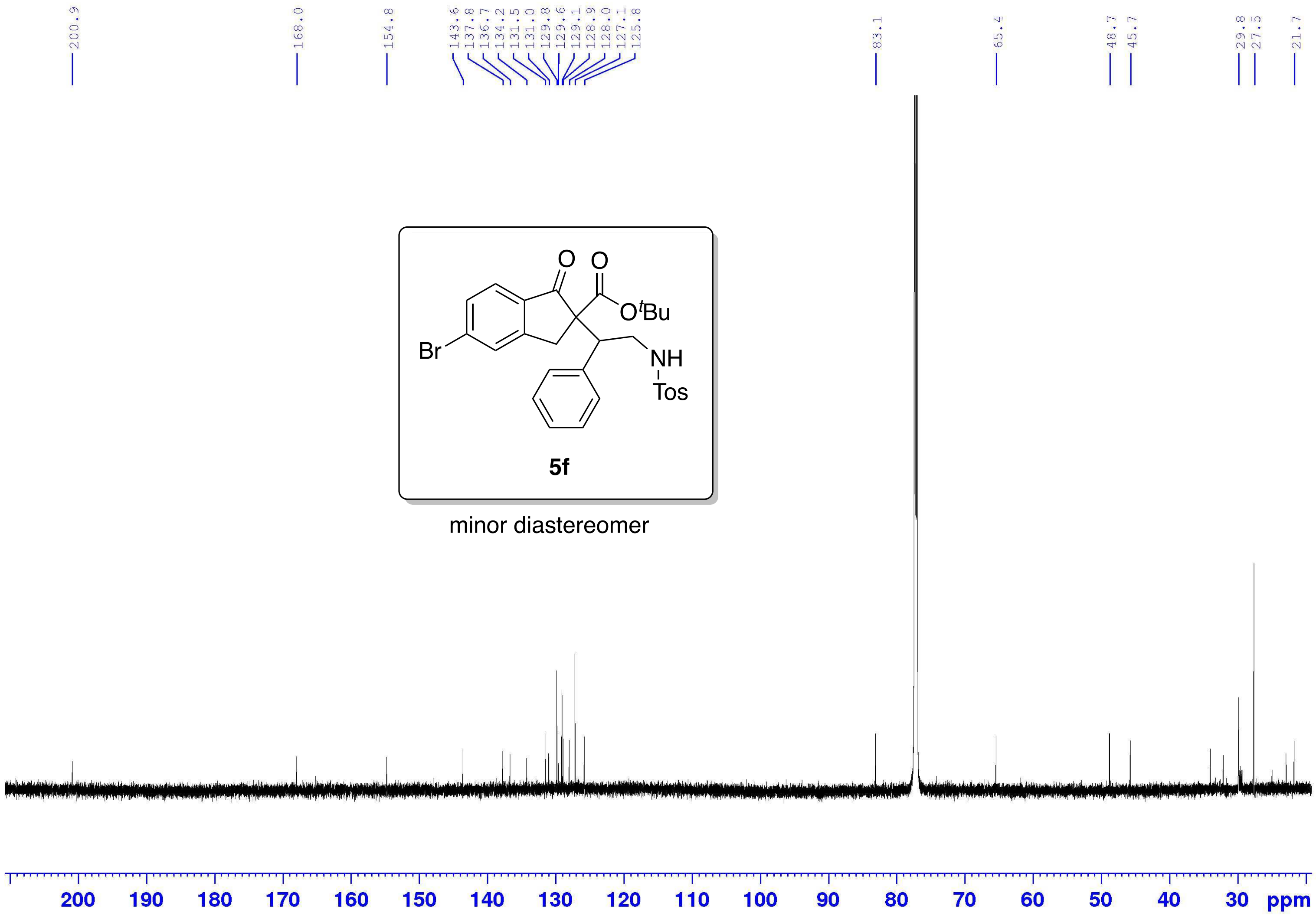
200.9
168.0
154.8
143.6
137.8
136.7
134.2
131.5
131.0
129.8
129.6
129.1
128.9
128.0
127.1
125.8
83.1
65.4
48.7
45.7
29.8
27.5
21.7
O
O
O^tBu
Br
NH
Tos
5f
minor diastereomer
200
190
180
170
160
150
140
130
120
110
100
90
80
70
60
50
40
30
ppm

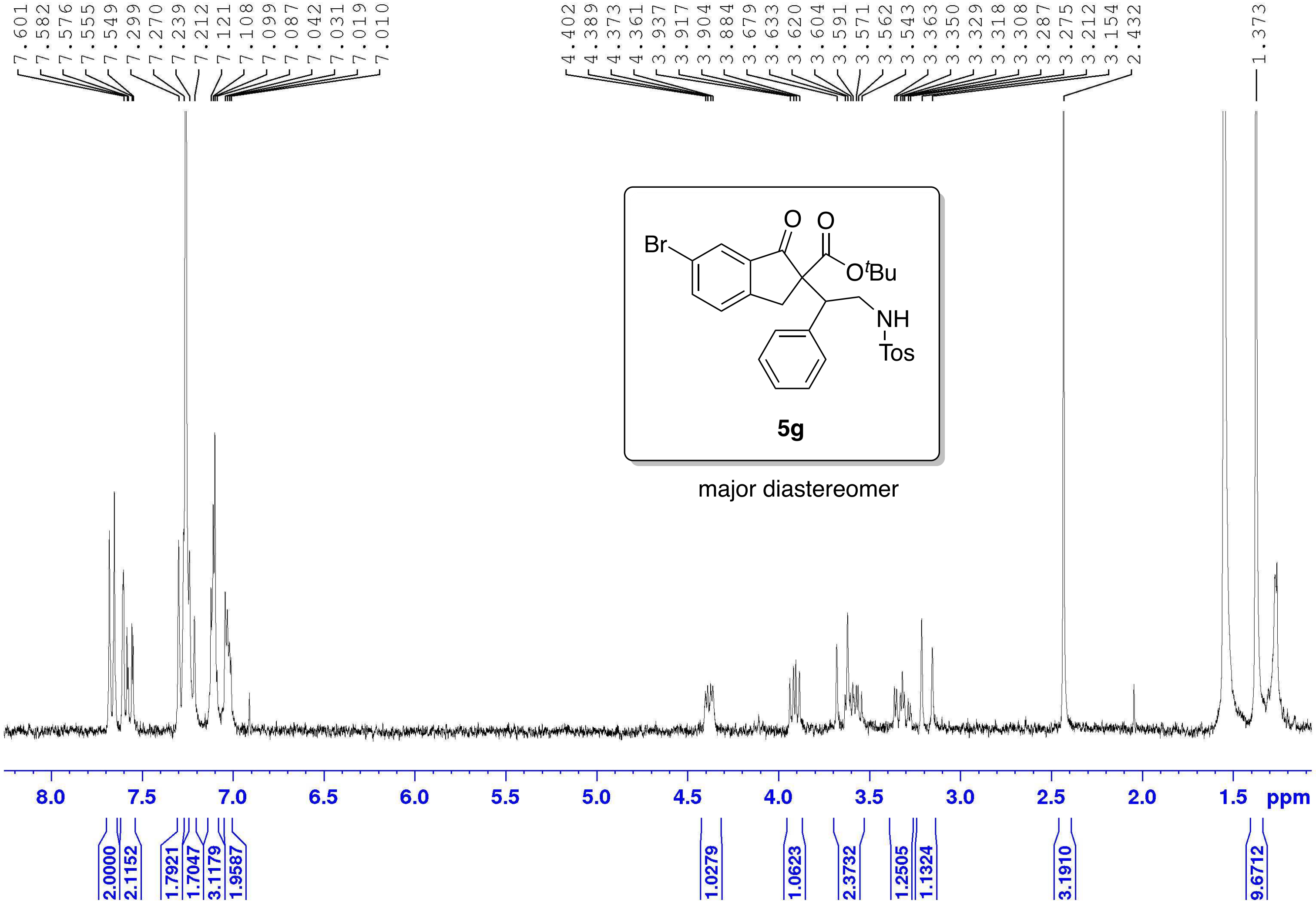
7.601
7.582
7.576
7.555
7.549
7.299
7.270
7.239
7.212
7.121
7.108
7.099
7.087
7.042
7.031
7.019
7.010
4.402
4.389
4.373
4.361
3.937
3.917
3.904
3.884
3.679
3.633
3.620
3.604
3.591
3.571
3.562
3.543
3.363
3.350
3.329
3.318
3.308
3.287
3.275
3.212
3.154
2.432
1.373
Br
O
O
O^tBu
NH
Tos
5g
major diastereomer
8.0
7.5
7.0
6.5
6.0
5.5
5.0
4.5
4.0
3.5
3.0
2.5
2.0
1.5
ppm
2.0000
2.1152
1.7921
1.7047
3.1179
1.9587
1.0279
1.0623
2.3732
1.2505
1.1324
3.1910
9.6712

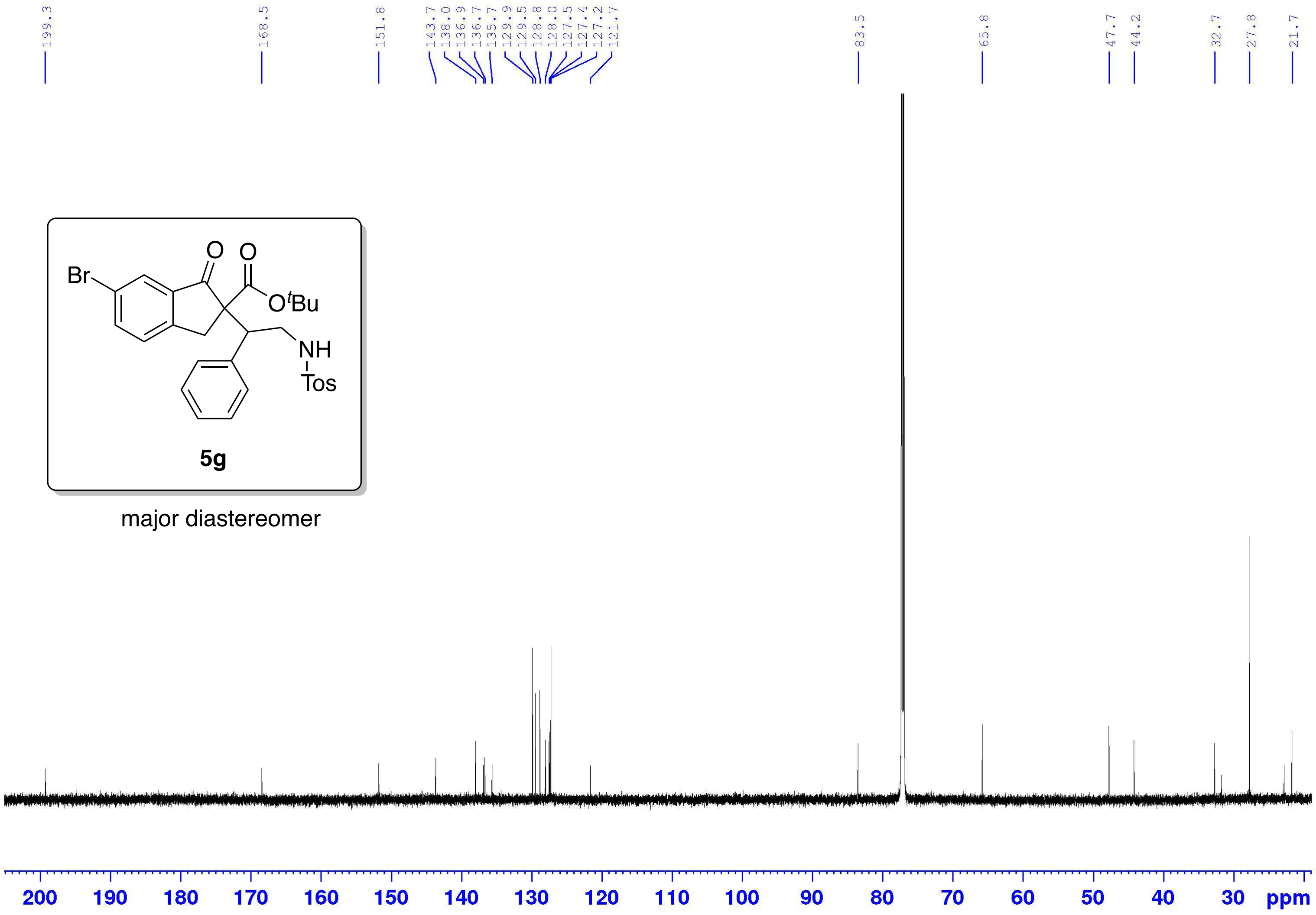
199.3
168.5
151.8
143.7
138.0
136.9
136.7
135.7
129.9
129.5
128.8
128.0
127.5
127.4
127.2
121.7
83.5
65.8
47.7
44.2
32.7
27.8
21.7
Br
O
O
O^{t}Bu
NH
Tos
5g
major diastereomer
200 190 180 170 160 150 140 130 120 110 100 90 80 70 60 50 40 30 ppm

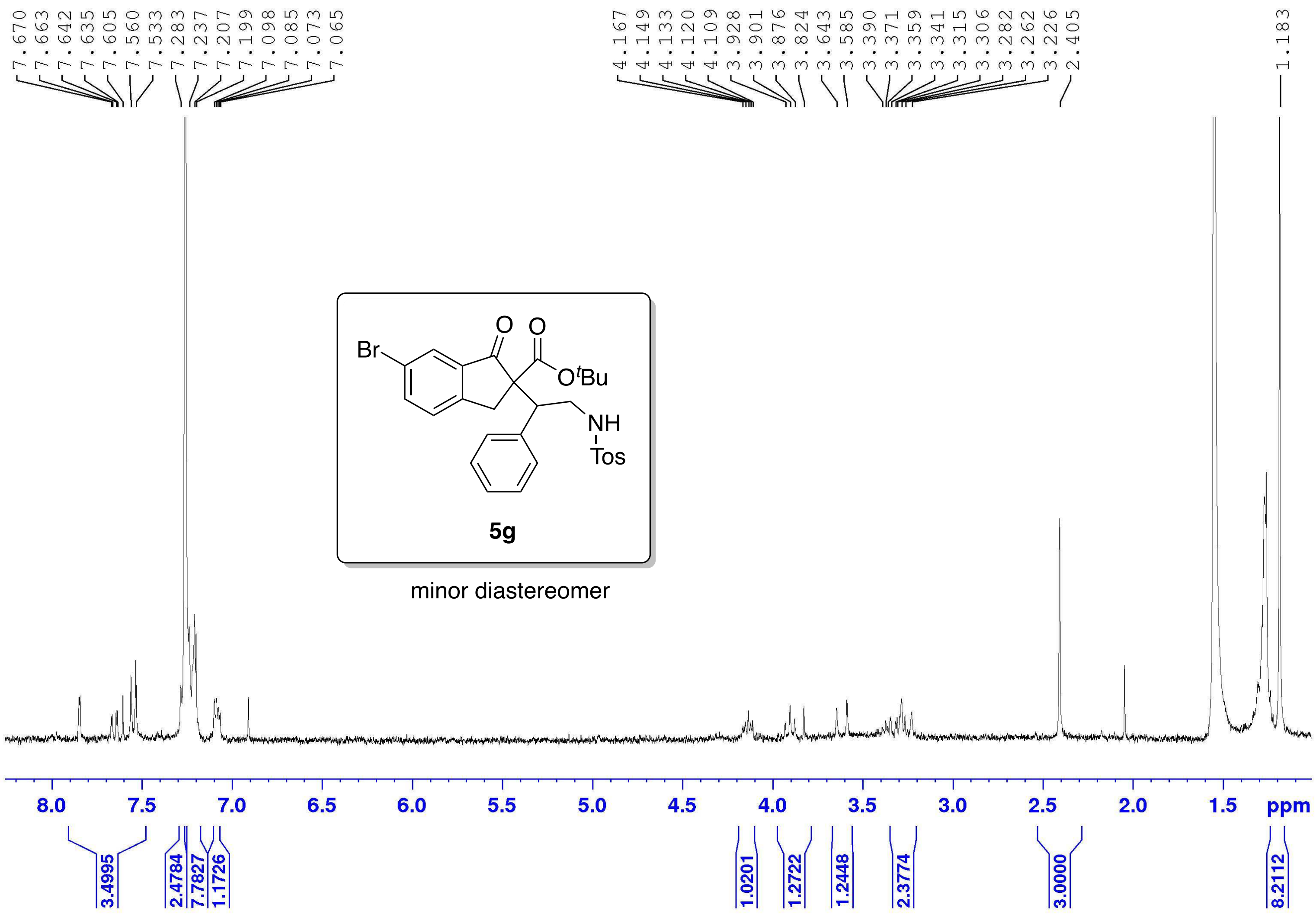
7.670
7.663
7.642
7.635
7.605
7.560
7.533
7.283
7.237
7.207
7.199
7.098
7.085
7.073
7.065
4.167
4.149
4.133
4.120
4.109
3.928
3.901
3.876
3.824
3.643
3.585
3.390
3.371
3.359
3.341
3.315
3.306
3.282
3.262
3.226
2.405
1.183
Br
O
O
O^{t}Bu
NH
Tos
5g
minor diastereomer
8.0
7.5
7.0
6.5
6.0
5.5
5.0
4.5
4.0
3.5
3.0
2.5
2.0
1.5
ppm
3.4995
2.4784
7.7827
1.1726
1.0201
1.2722
1.2448
2.3774
3.0000
8.2112

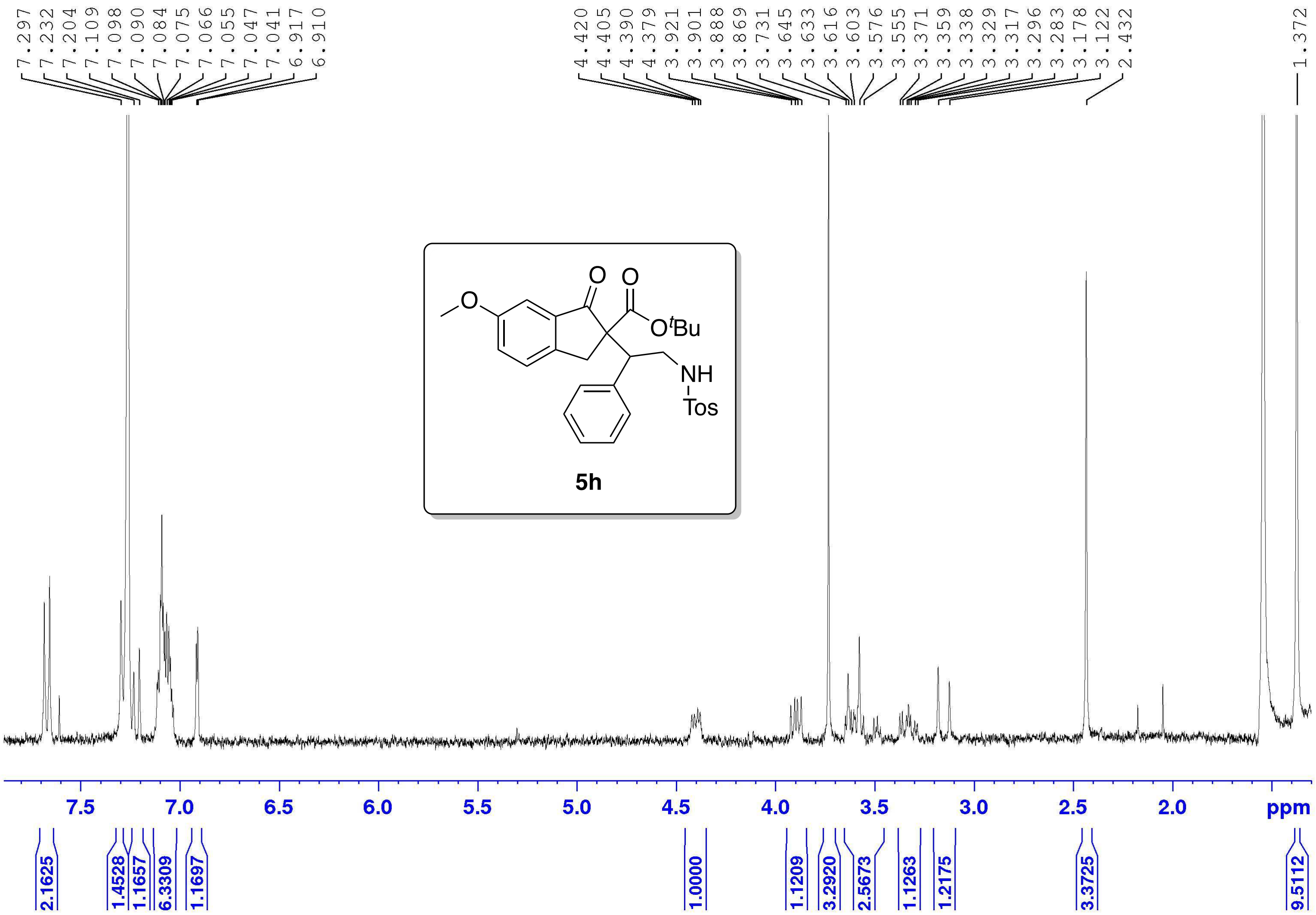
7.297 7.232 7.204 7.109 7.098 7.090 7.084 7.075 7.066 7.055 7.047 7.041 6.917 6.910
4.420 4.405 4.390 4.379 3.921 3.901 3.888 3.869 3.731 3.645 3.633 3.616 3.603 3.576 3.555 3.371 3.359 3.338 3.329 3.317 3.296 3.283 3.178 3.122 2.432
1.372
O
O
O^{t}Bu
NH
Tos
5h
7.5 7.0 6.5 6.0 5.5 5.0 4.5 4.0 3.5 3.0 2.5 2.0 ppm
2.1625 1.4528 1.1657 6.3309 1.1697 1.0000 1.1209 3.2920 2.5673 1.1263 1.2175 3.3725 9.5112

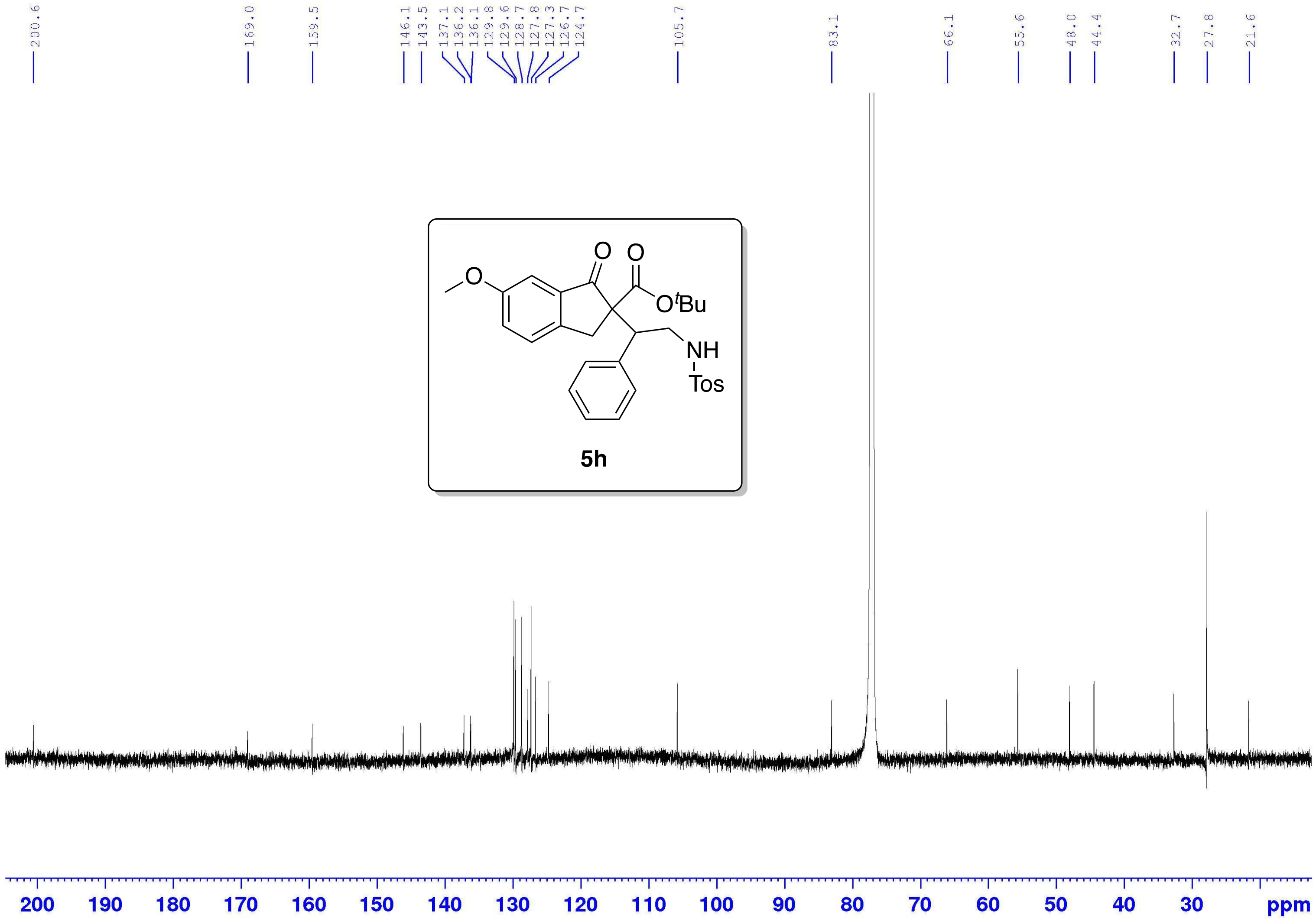
200.6
169.0
159.5
146.1
143.5
137.1
136.2
136.1
129.8
129.6
128.7
127.8
127.3
126.7
124.7
105.7
83.1
66.1
55.6
48.0
44.4
32.7
27.8
21.6
O
O
O
O^{t}Bu
NH
Tos
5h
200
190
180
170
160
150
140
130
120
110
100
90
80
70
60
50
40
30
ppm

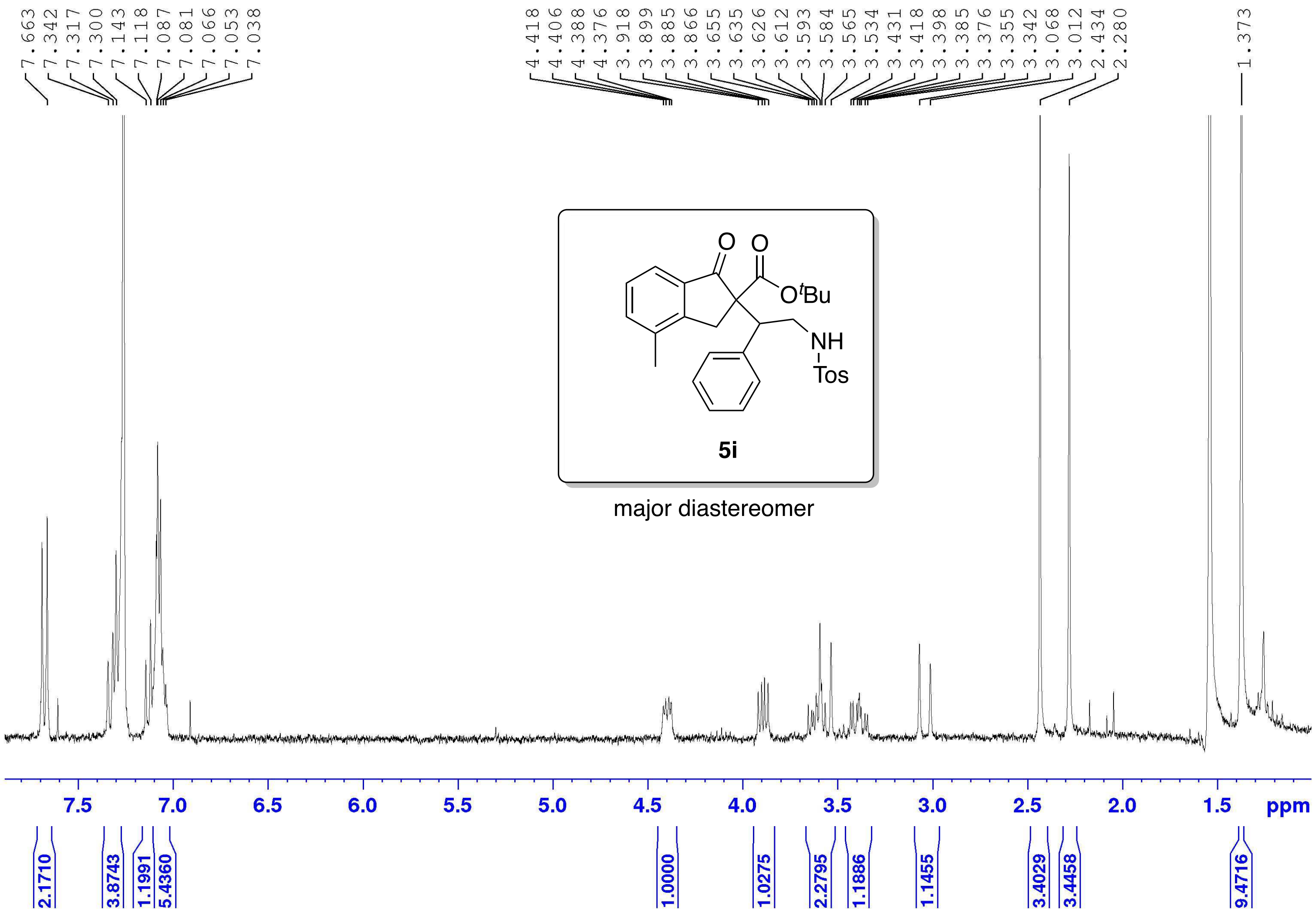
7.663 7.342 7.317 7.300 7.143 7.118 7.087 7.081 7.066 7.053 7.038
4.418 4.406 4.388 4.376 3.918 3.899 3.885 3.866 3.655 3.635 3.626 3.612 3.593 3.584 3.565 3.534 3.431 3.418 3.398 3.385 3.376 3.355 3.342 3.068 3.012 2.434 2.280
1.373
O
O
O^{t}Bu
NH
Tos
5i
major diastereomer
7.5 7.0 6.5 6.0 5.5 5.0 4.5 4.0 3.5 3.0 2.5 2.0 1.5 ppm
2.1710 3.8743 1.1991 5.4360 1.0000 1.0275 2.2795 1.1886 1.1455 3.4029 3.4458 9.4716

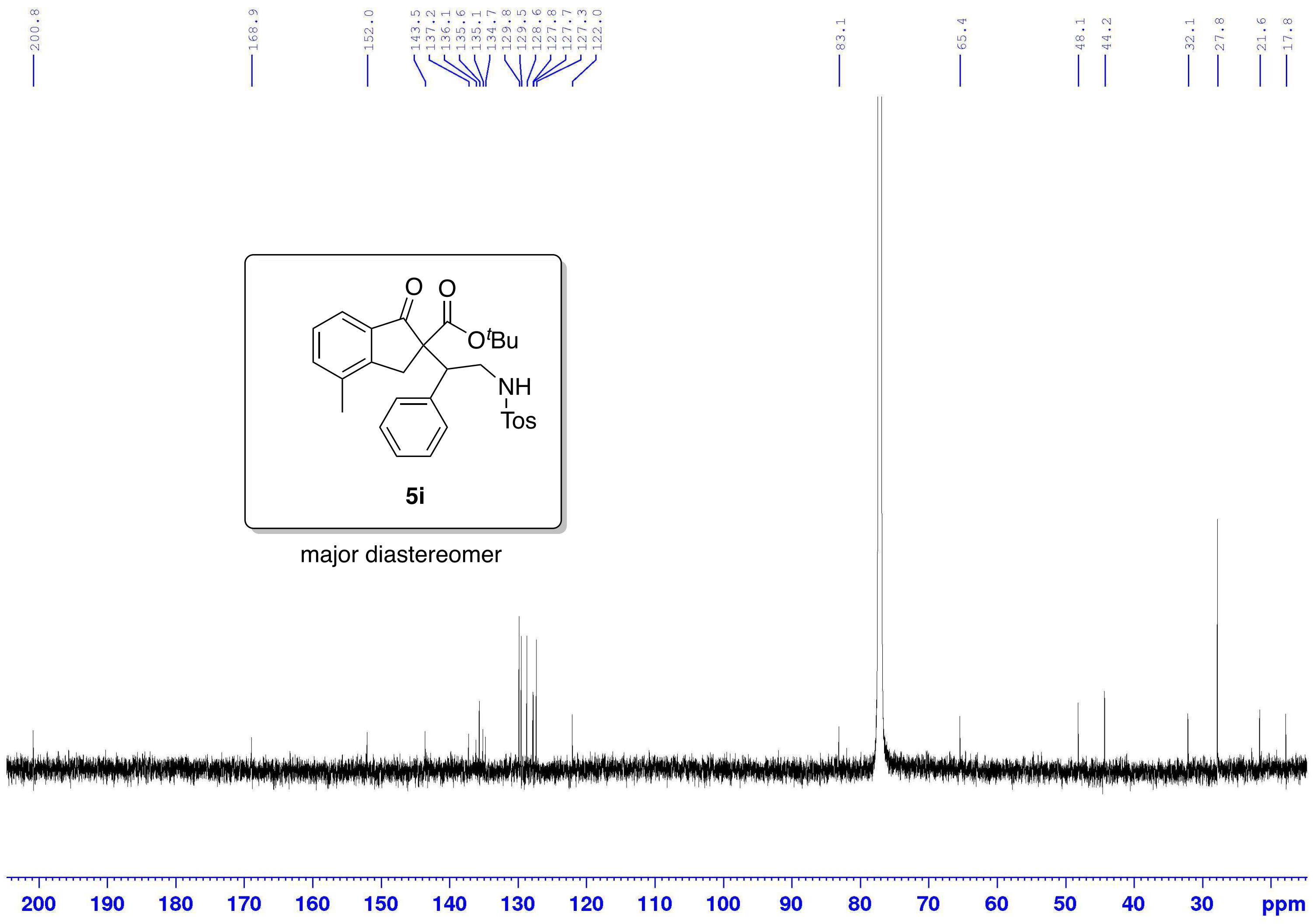
200.8
168.9
152.0
143.5
137.2
136.1
135.6
135.1
134.7
129.8
129.5
128.6
127.8
127.7
127.3
122.0
83.1
65.4
48.1
44.2
32.1
27.8
21.6
17.8
O
O
O^{t}Bu
NH
Tos
5i
major diastereomer
200
190
180
170
160
150
140
130
120
110
100
90
80
70
60
50
40
30
ppm

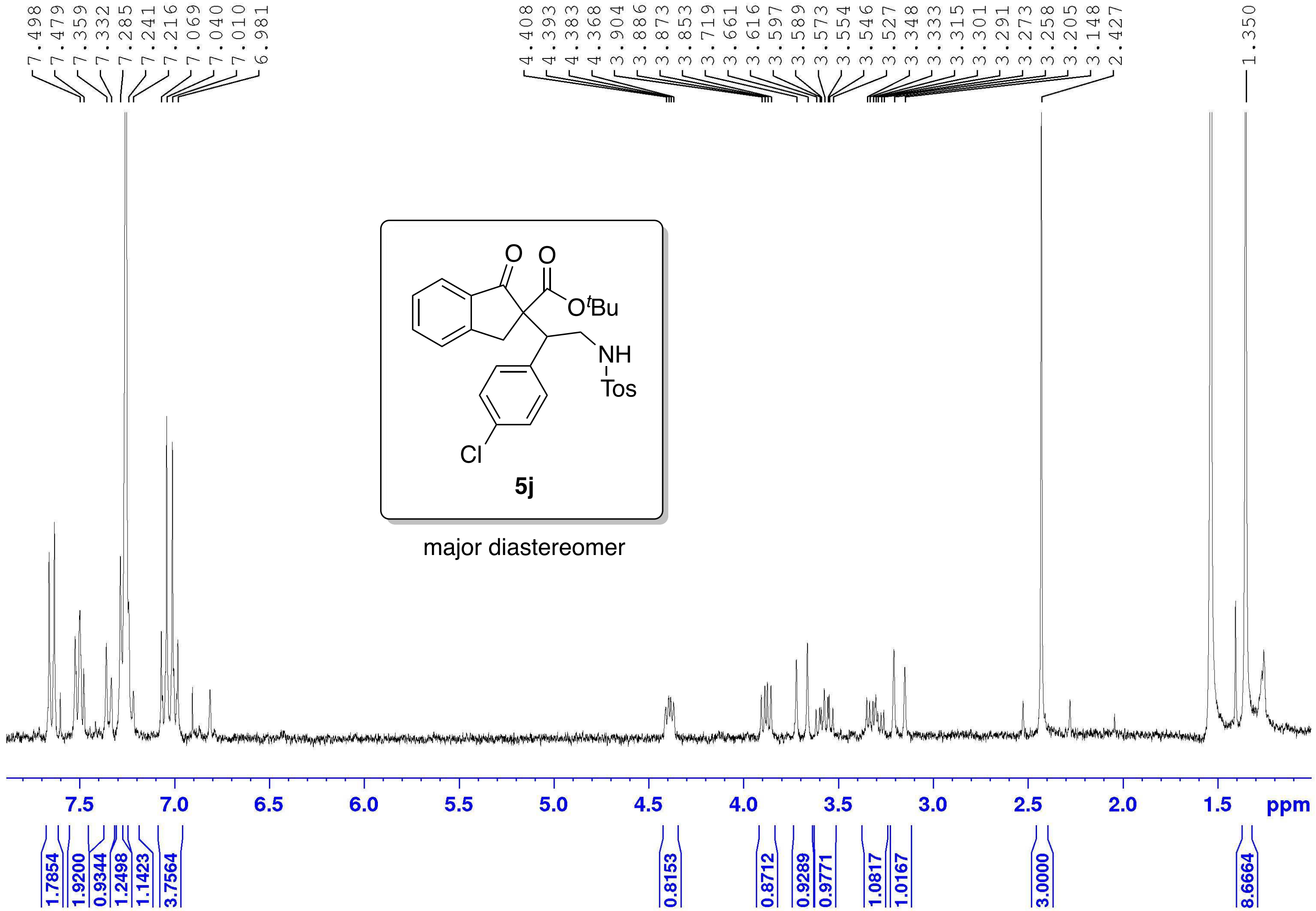
7.498
7.479
7.359
7.332
7.285
7.241
7.216
7.069
7.040
7.010
6.981
4.408
4.393
4.383
4.368
3.904
3.886
3.873
3.853
3.719
3.661
3.616
3.597
3.589
3.573
3.554
3.546
3.527
3.348
3.333
3.315
3.301
3.291
3.273
3.258
3.205
3.148
2.427
1.350
O
O
O^{t}Bu
NH
Tos
Cl
5j
major diastereomer
7.5
7.0
6.5
6.0
5.5
5.0
4.5
4.0
3.5
3.0
2.5
2.0
1.5
ppm
1.7854
1.9200
0.9344
1.2498
1.1423
3.7564
0.8153
0.8712
0.9289
0.9771
1.0817
1.0167
3.0000
8.6664

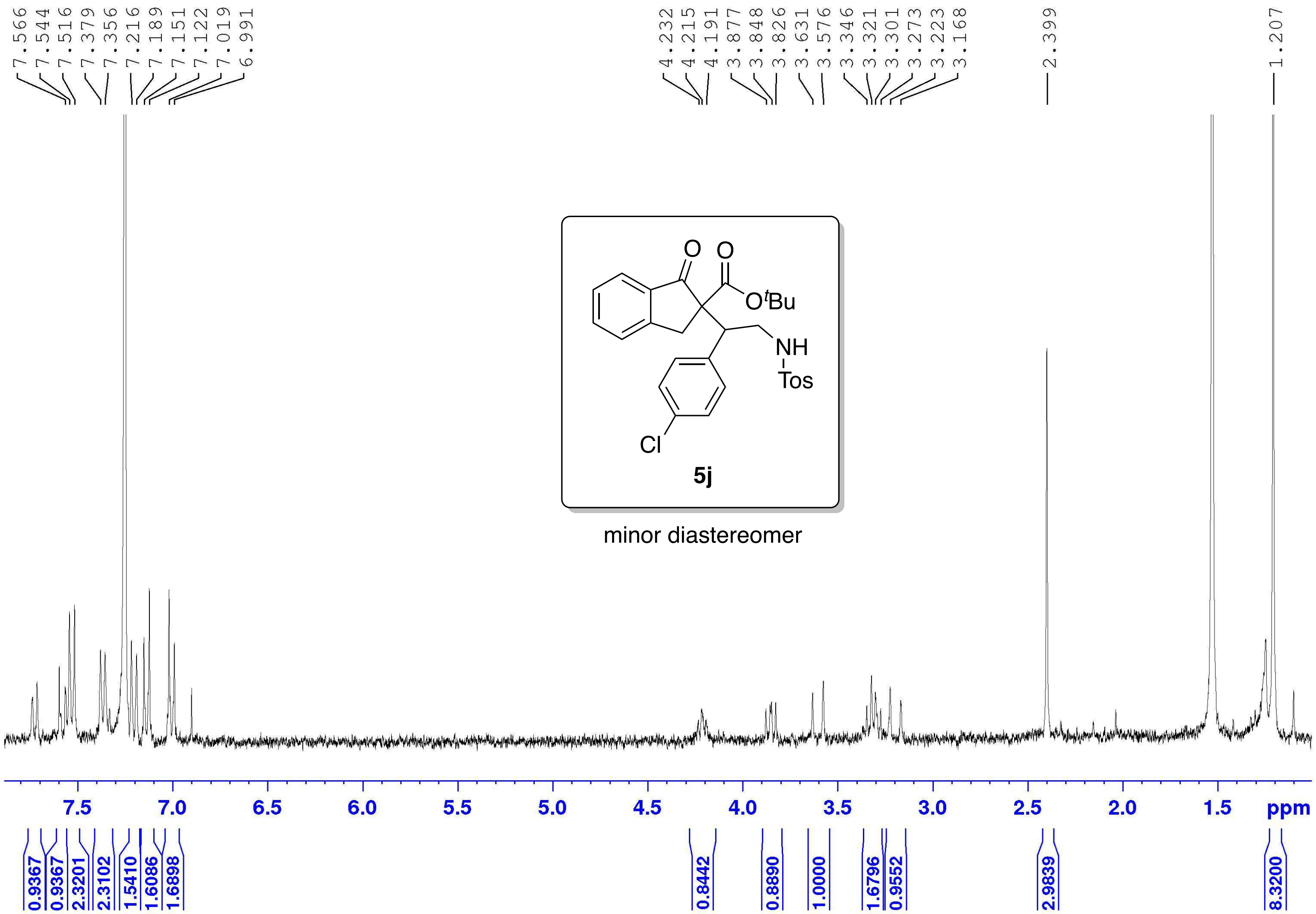
7.566
7.544
7.516
7.379
7.356
7.216
7.189
7.151
7.122
7.019
6.991
4.232
4.215
4.191
3.877
3.848
3.826
3.631
3.576
3.346
3.321
3.301
3.273
3.223
3.168
2.399
1.207
O
O
O^tBu
NH
Tos
Cl
5j
minor diastereomer
7.5
7.0
6.5
6.0
5.5
5.0
4.5
4.0
3.5
3.0
2.5
2.0
1.5
ppm
0.9367
0.9367
2.3201
2.3102
1.5410
1.6086
1.6898
0.8442
0.8890
1.0000
1.6796
0.9552
2.9839
8.3200

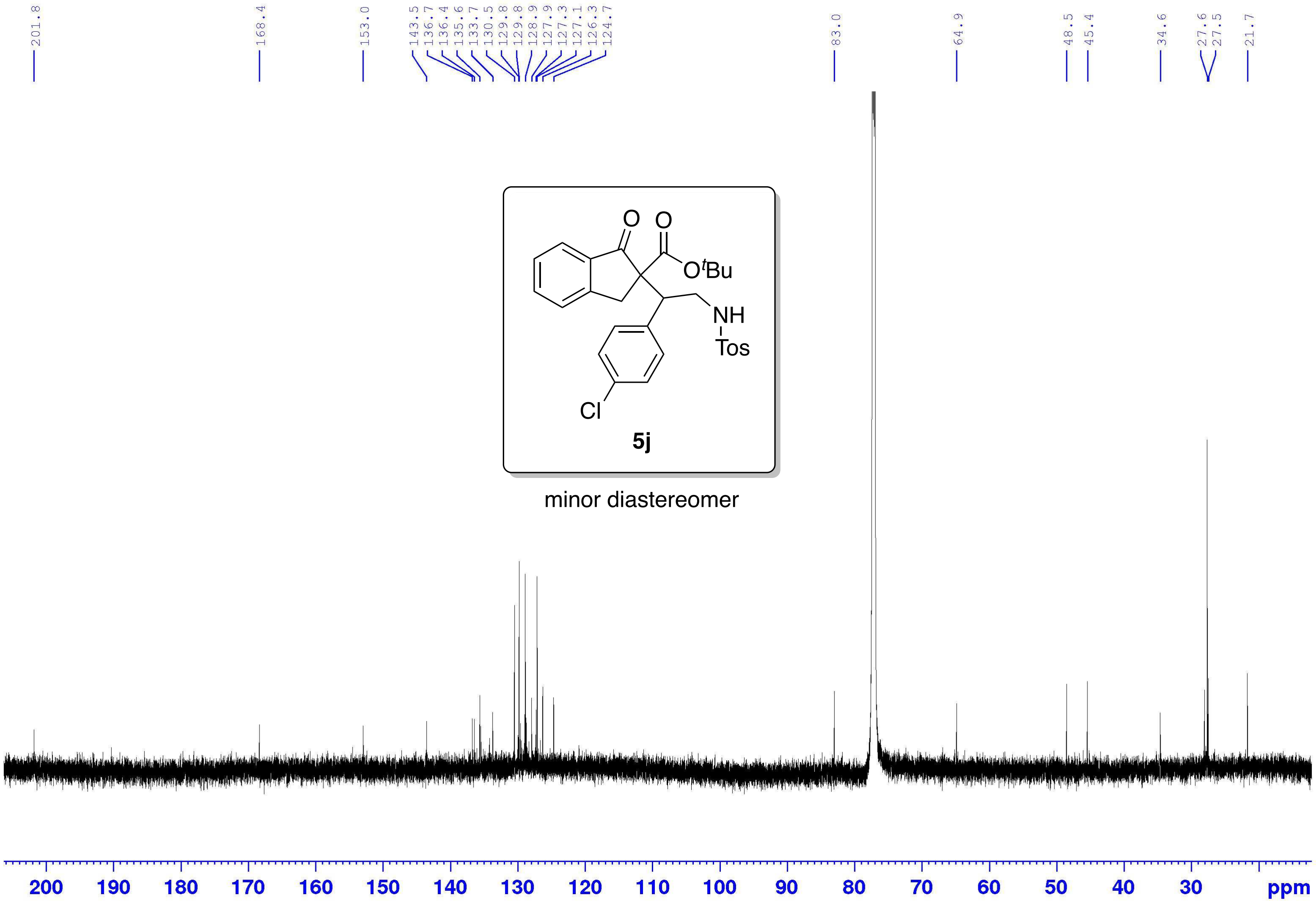
201.8
168.4
153.0
143.5
136.7
136.4
135.6
133.7
130.5
129.8
129.8
128.9
127.9
127.3
127.1
126.3
124.7
83.0
64.9
48.5
45.4
34.6
27.6
27.5
21.7
O
O
O^{t}Bu
NH
Tos
Cl
5j
minor diastereomer
200
190
180
170
160
150
140
130
120
110
100
90
80
70
60
50
40
30
ppm

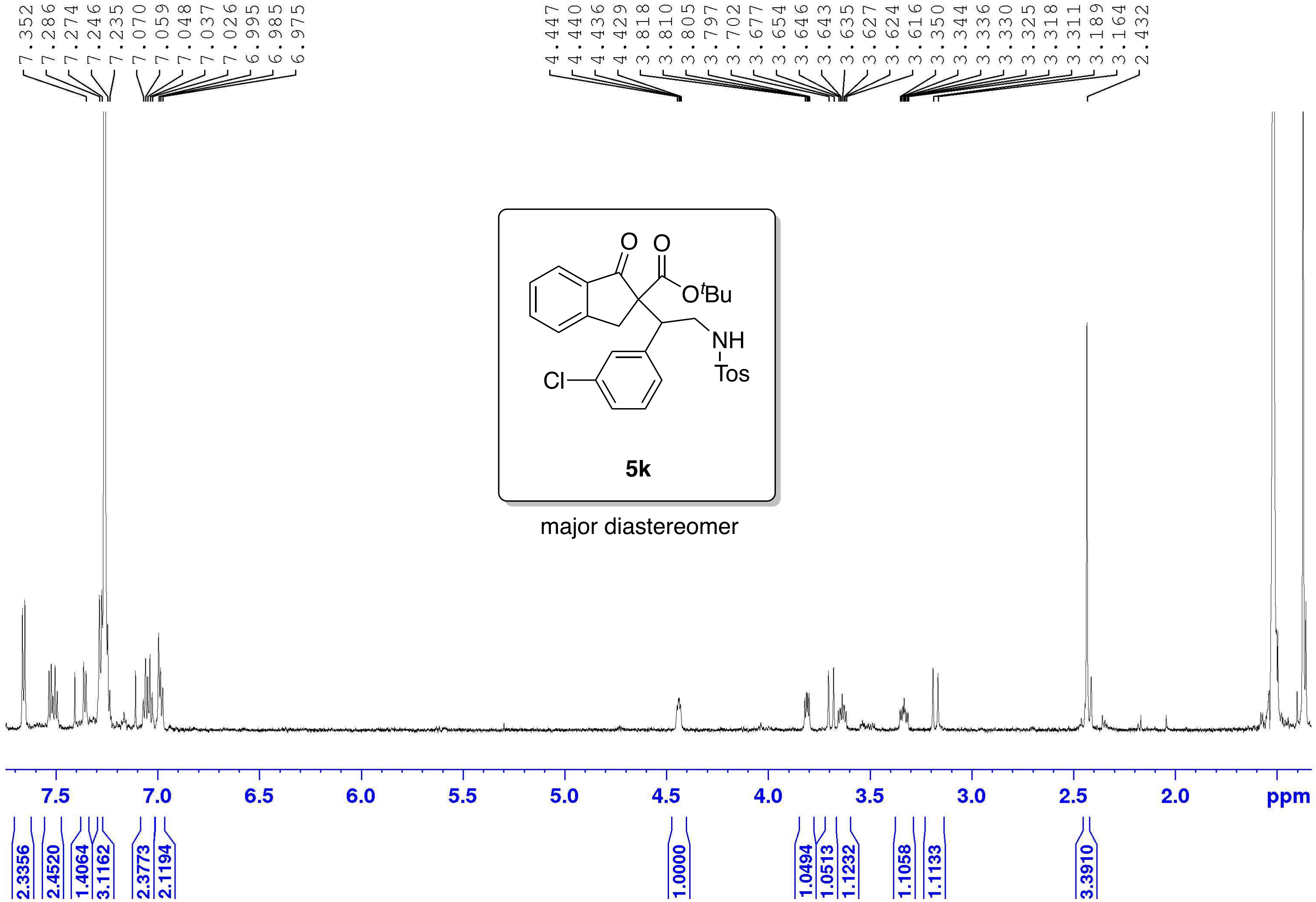
7.352
7.286
7.274
7.246
7.235
7.070
7.059
7.048
7.037
7.026
6.995
6.985
6.975
4.447
4.440
4.436
4.429
3.818
3.810
3.805
3.797
3.702
3.677
3.654
3.646
3.643
3.635
3.627
3.624
3.616
3.350
3.344
3.336
3.330
3.325
3.318
3.311
3.189
3.164
2.432
O
O
O^{t}Bu
NH
Tos
Cl
5k
major diastereomer
7.5
7.0
6.5
6.0
5.5
5.0
4.5
4.0
3.5
3.0
2.5
2.0
ppm
2.3356
2.4520
1.4064
3.1162
2.3773
2.1194
1.0000
1.0494
1.0513
1.1232
1.1058
1.1133
3.3910

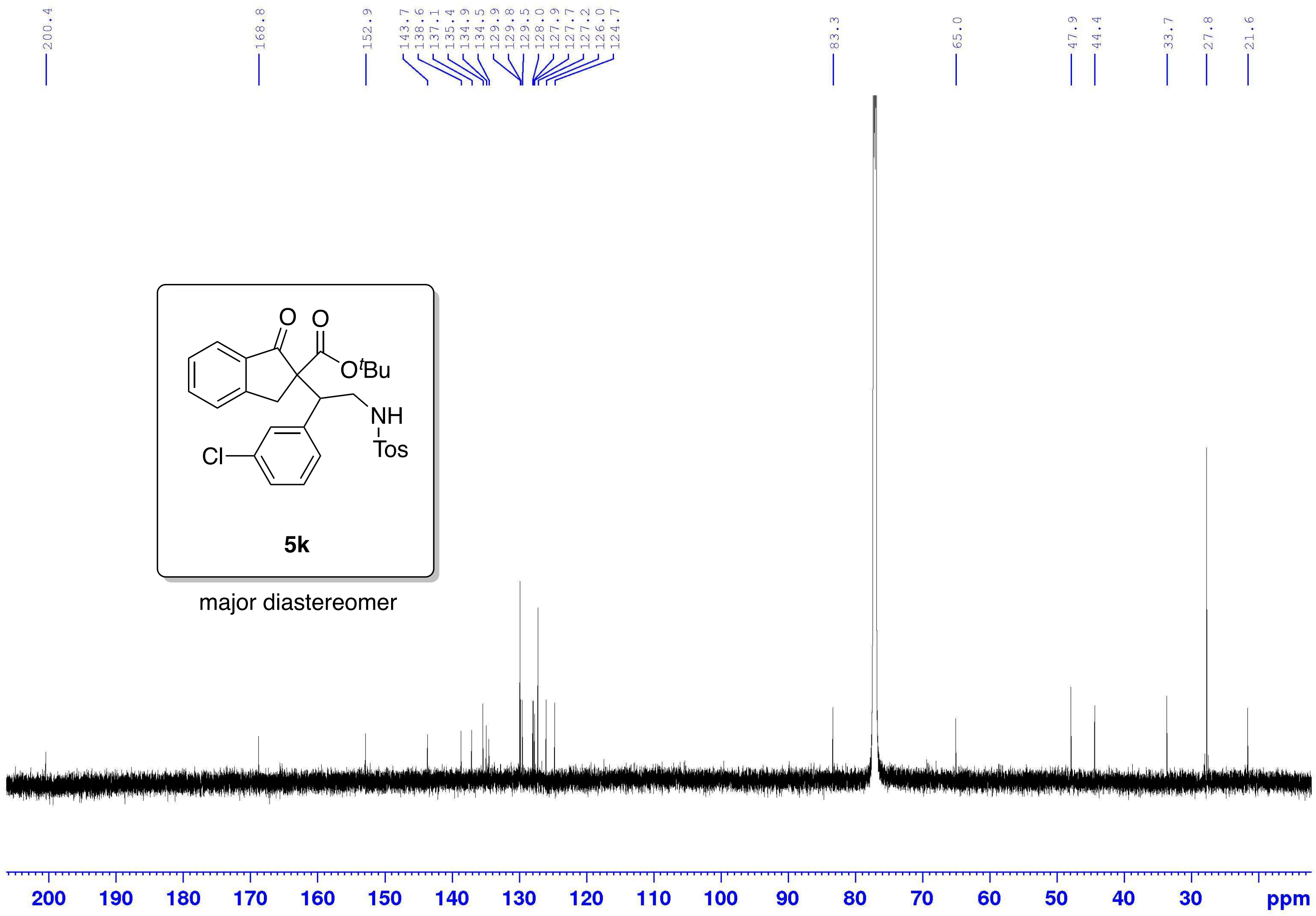
200.4
168.8
152.9
143.7
138.6
137.1
135.4
134.9
134.5
129.9
129.8
129.5
128.0
127.9
127.7
127.2
126.0
124.7
83.3
65.0
47.9
44.4
33.7
27.8
21.6
O
O
O^tBu
NH
Tos
Cl
5k
major diastereomer
200 190 180 170 160 150 140 130 120 110 100 90 80 70 60 50 40 30 ppm

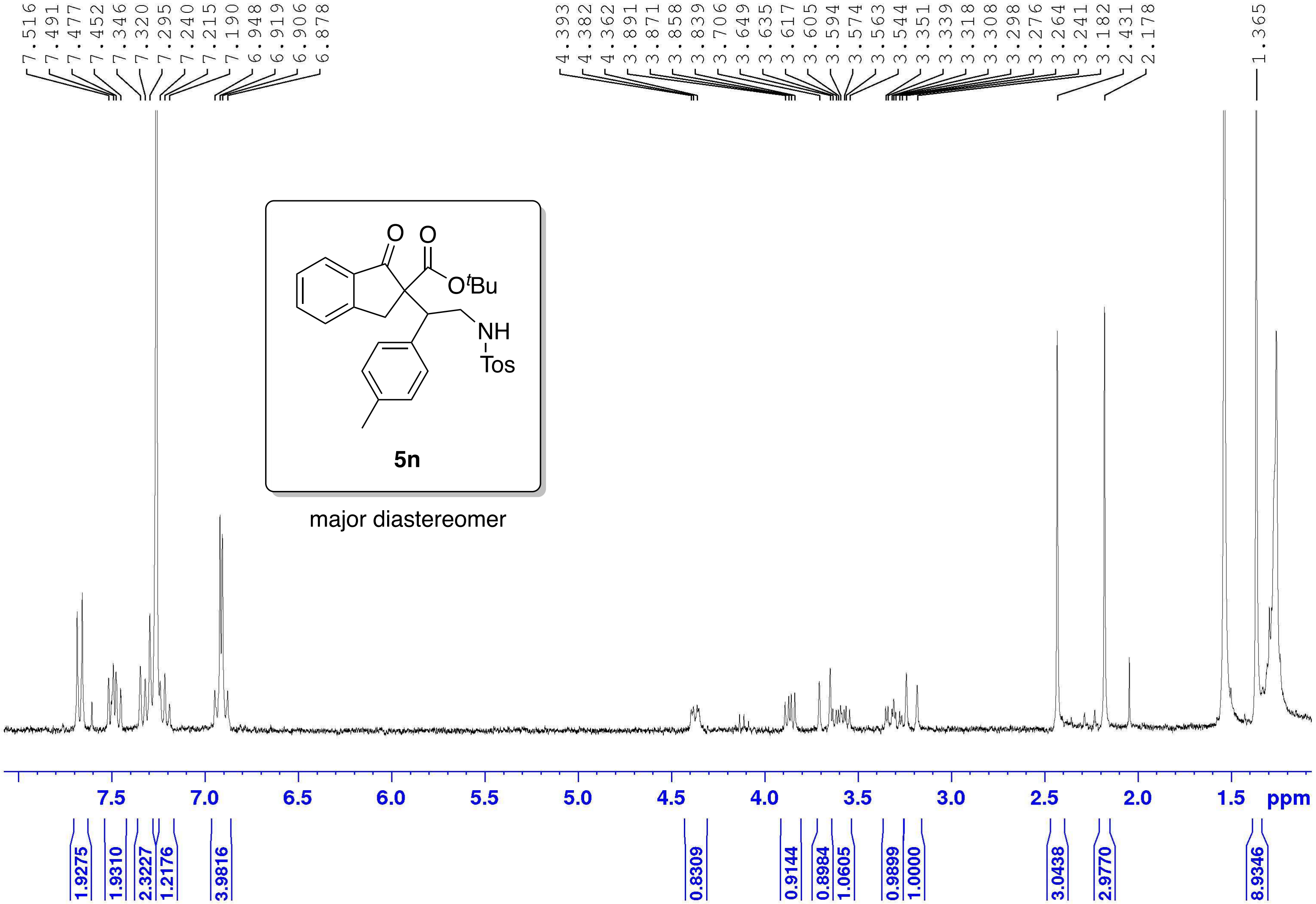
7.516 7.491 7.477 7.452 7.346 7.320 7.295 7.240 7.215 7.190 6.948 6.919 6.906 6.878
4.393 4.382 4.362 3.891 3.871 3.858 3.839 3.706 3.649 3.635 3.617 3.605 3.594 3.574 3.563 3.544 3.351 3.339 3.318 3.308 3.298 3.276 3.264 3.241 3.182 2.431 2.178
1.365
O
O
O^{t}Bu
NH
Tos
5n
major diastereomer
7.5 7.0 6.5 6.0 5.5 5.0 4.5 4.0 3.5 3.0 2.5 2.0 1.5 ppm
1.9275 1.9310 2.3227 1.2176 3.9816 0.8309 0.9144 0.8984 1.0605 0.9899 1.0000 3.0438 2.9770 8.9346

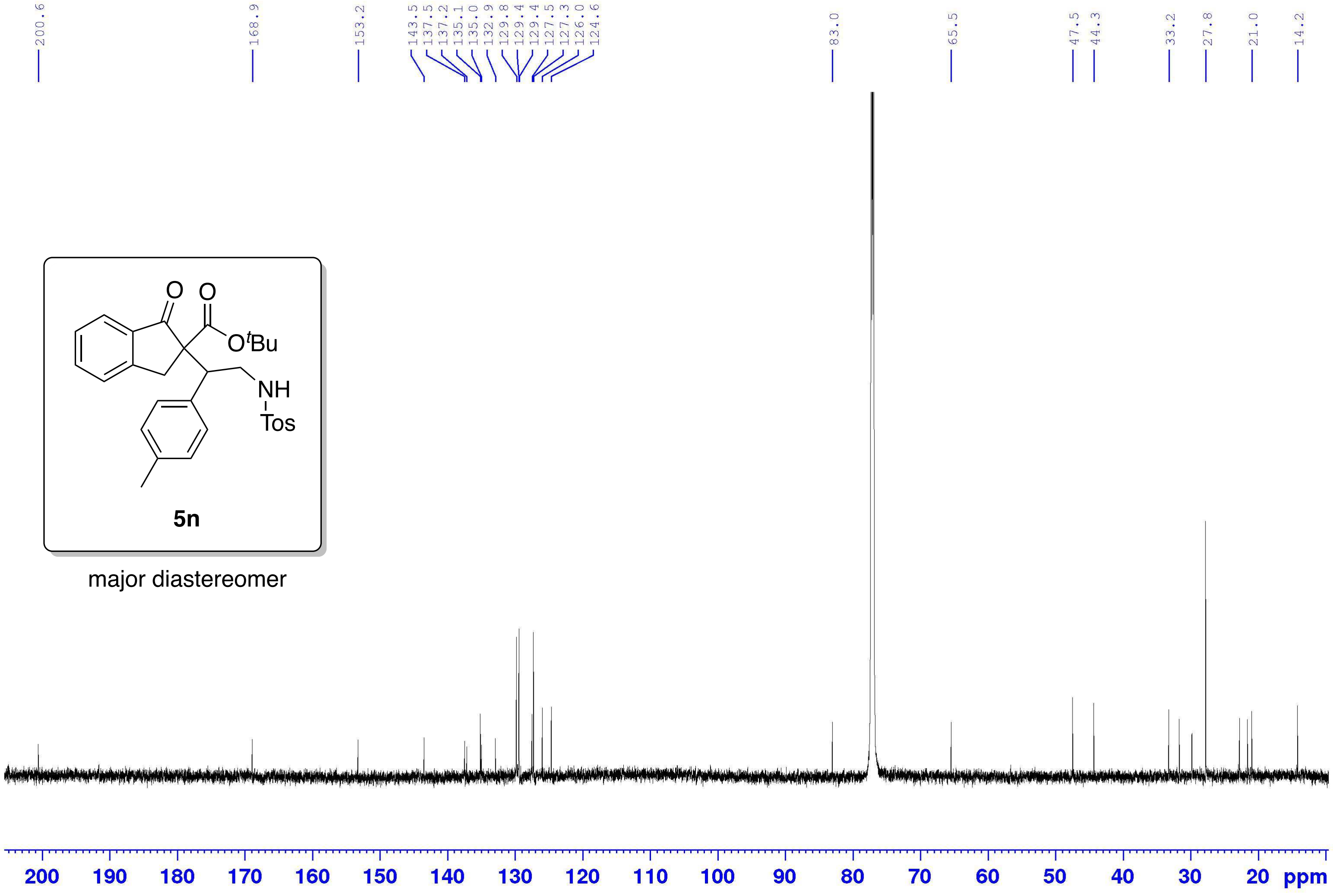

major diastereomer

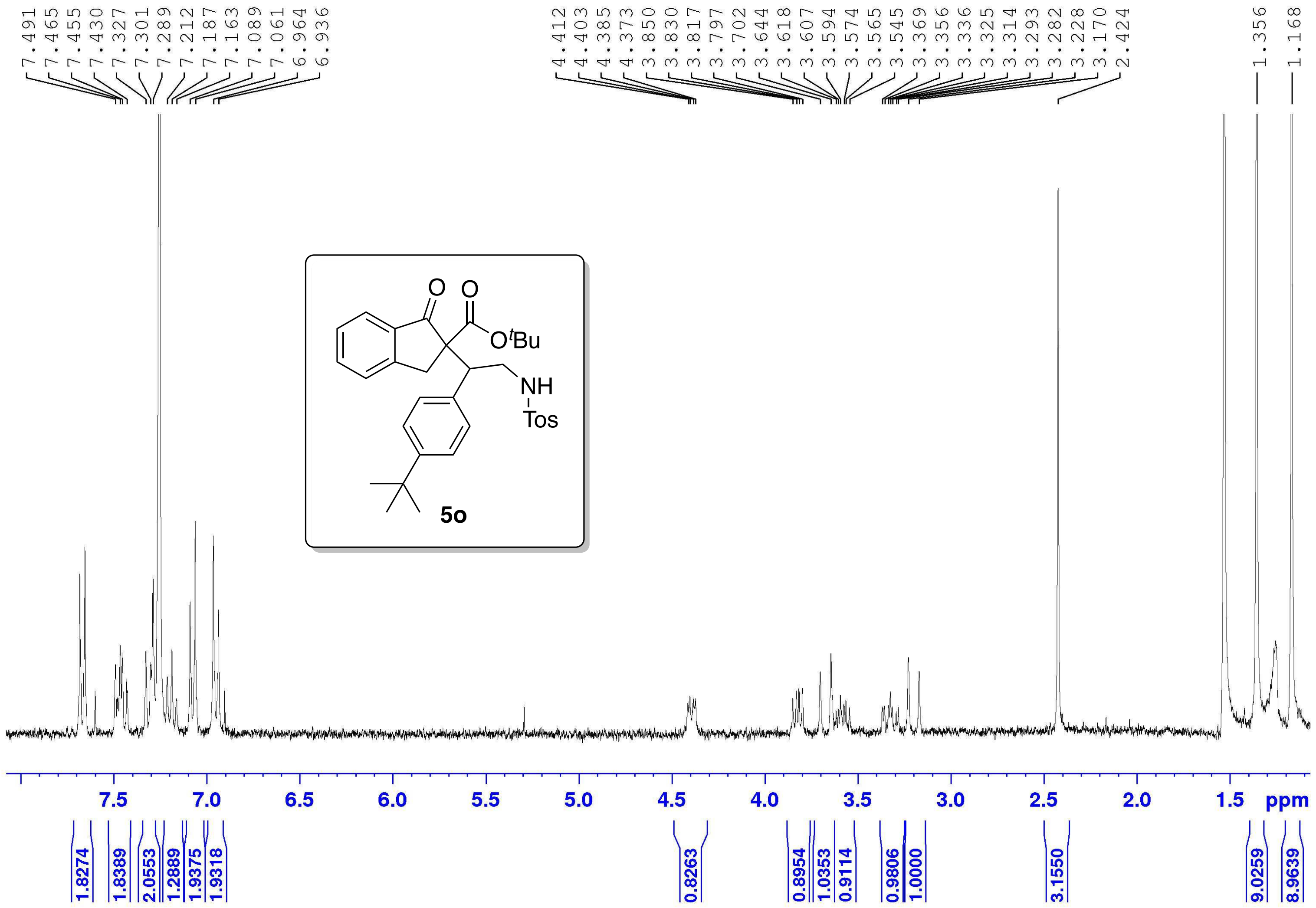
7.491
7.465
7.455
7.430
7.327
7.301
7.289
7.212
7.187
7.163
7.089
7.061
6.964
6.936
4.412
4.403
4.385
4.373
3.850
3.830
3.817
3.797
3.702
3.644
3.618
3.607
3.594
3.574
3.565
3.545
3.369
3.356
3.336
3.325
3.314
3.293
3.282
3.228
3.170
2.424
1.356
1.168
O
O
O^{t}Bu
NH
Tos
5o
7.5
7.0
6.5
6.0
5.5
5.0
4.5
4.0
3.5
3.0
2.5
2.0
1.5
ppm
1.8274
1.8389
2.0553
1.2889
1.9375
1.9318
0.8263
0.8954
1.0353
0.9114
0.9806
1.0000
3.1550
9.0259
8.9639

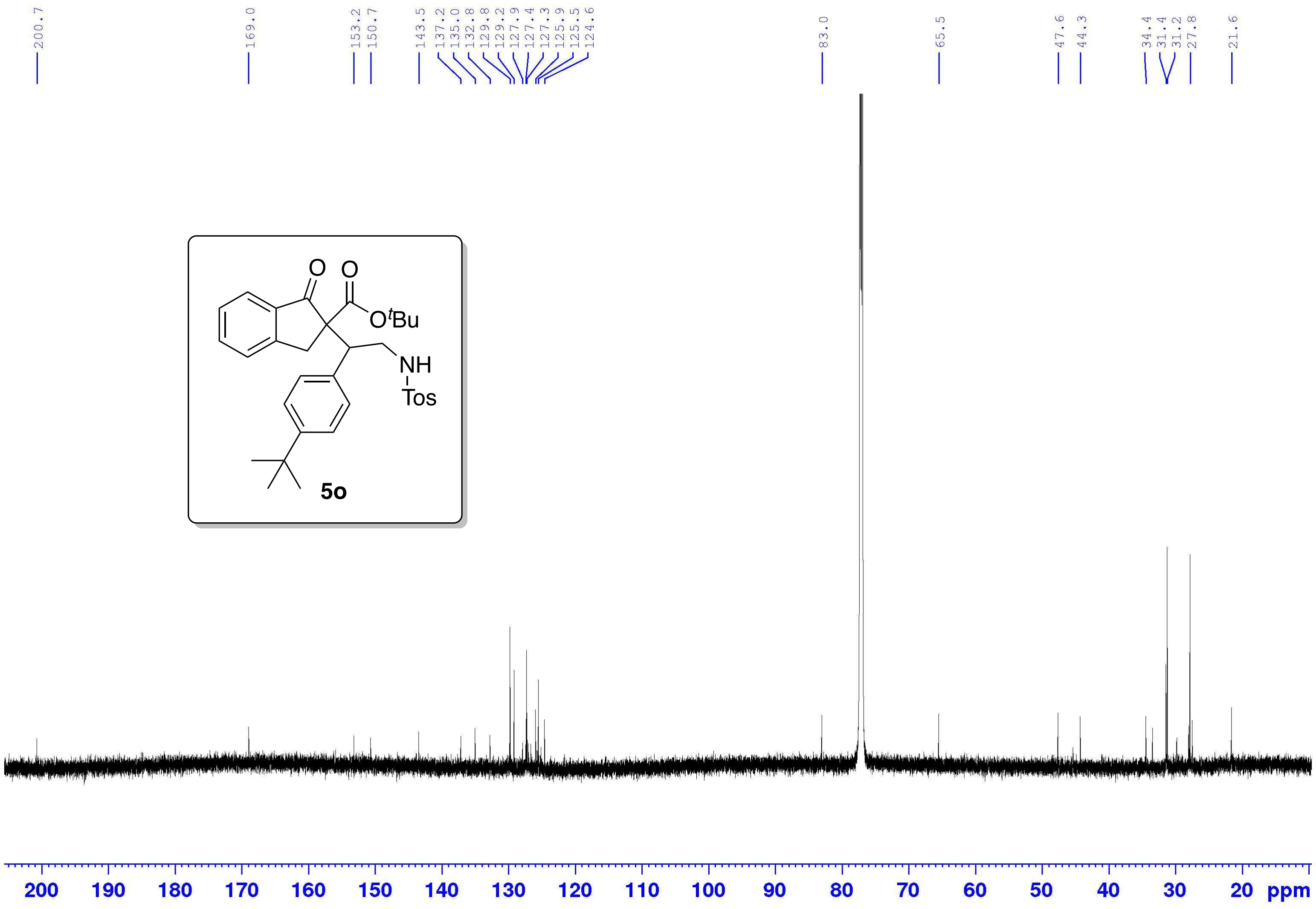
200.7
169.0
153.2
150.7
143.5
137.2
135.0
132.8
129.8
129.2
127.9
127.4
127.3
125.9
125.5
124.6
83.0
65.5
47.6
44.3
34.4
31.4
31.2
27.8
21.6
O
O
O^{t}Bu
NH
Tos
5o
200 190 180 170 160 150 140 130 120 110 100 90 80 70 60 50 40 30 20 ppm

4. HPLC Traces

Determination of the diastereomeric ratio of racemic **5a** by HPLC (YMC-SA)

21.04 min: minor diastereomer, first enantiomer

26.61 min: major diastereomer, both enantiomers

35.45 min: minor diastereomer, second enantiomer

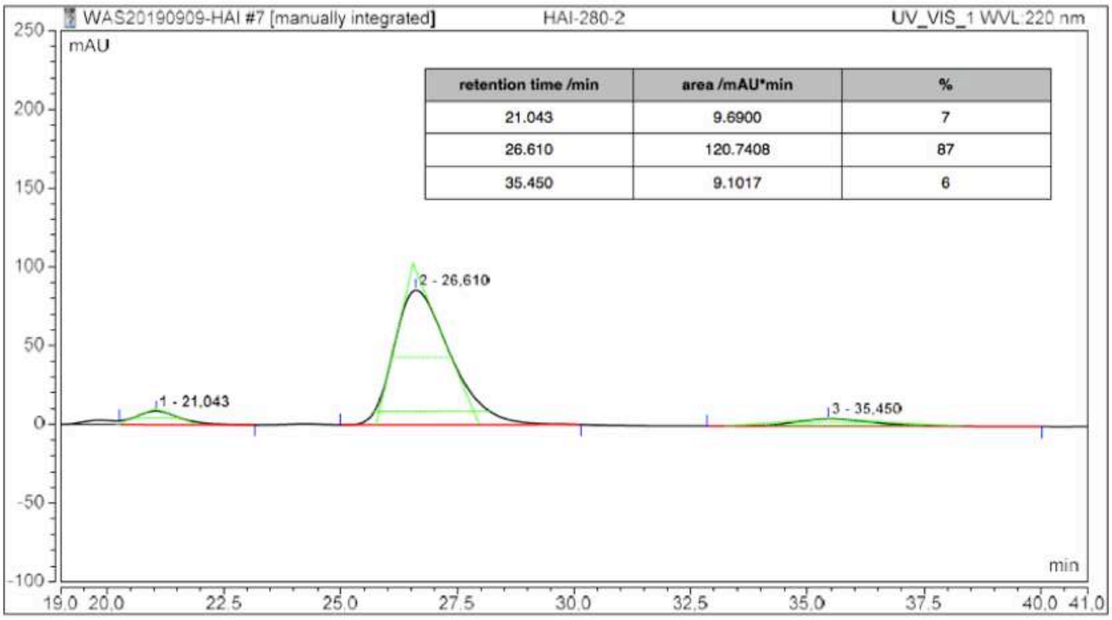

Product **5a** major diastereomer (racemic mixture)

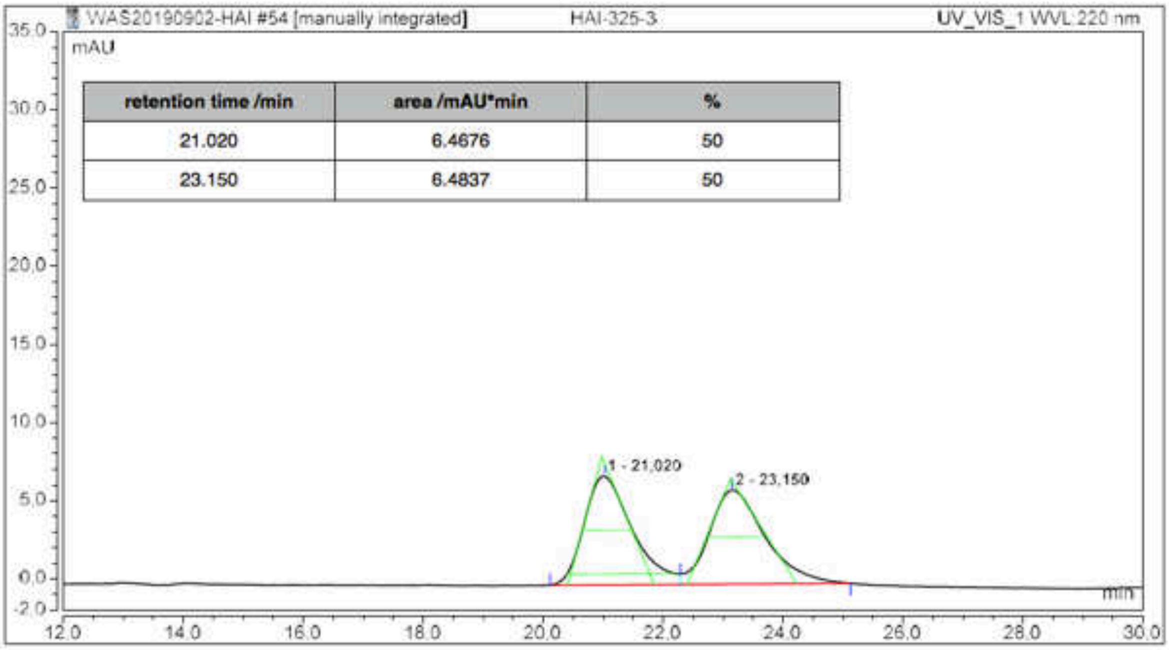

retention time /min	area /mAU*min	%
21.020	6.4676	50
23.150	6.4837	50

Product **5a** major diastereomer (enantiomer-enriched mixture)

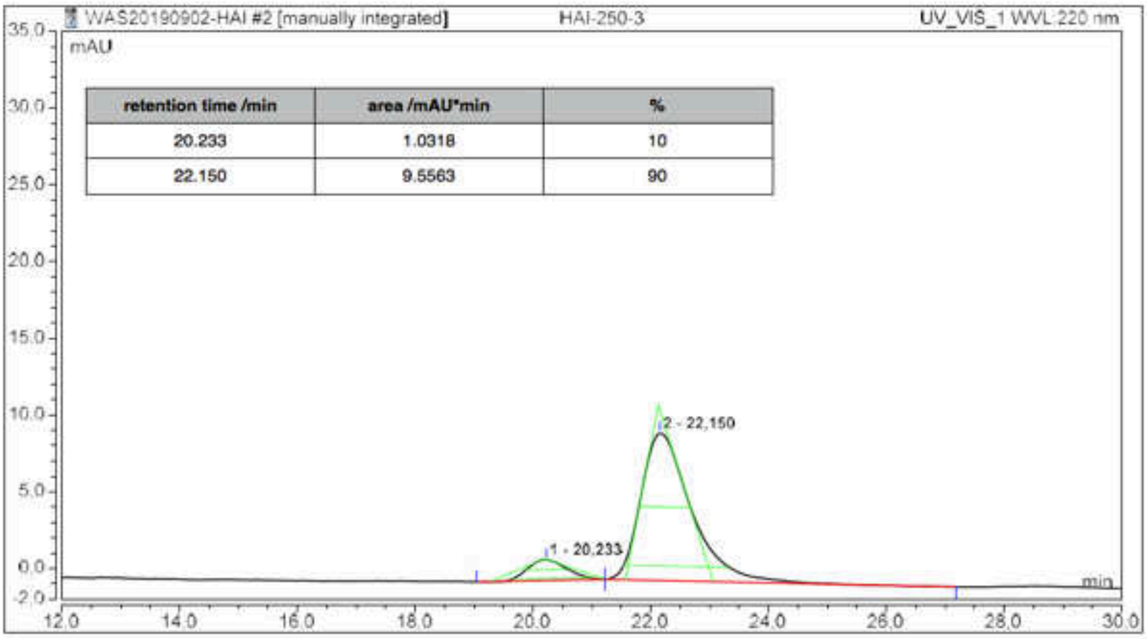

retention time /min	area /mAU*min	%
20.233	1.0318	10
22.150	9.5563	90
